# Bioassay- and metabolomics-guided screening of bioactive soil actinomycetes from the ancient city of Ihnasia, Egypt

**DOI:** 10.1371/journal.pone.0226959

**Published:** 2019-12-30

**Authors:** Mohamed Sebak, Amal E. Saafan, Sameh AbdelGhani, Walid Bakeer, Ahmed O. El-Gendy, Laia Castaño Espriu, Katherine Duncan, RuAngelie Edrada-Ebel

**Affiliations:** 1 Strathclyde Institute of Pharmacy and Biomedical Sciences, Faculty of Science, University of Strathclyde, Glasgow, Scotland, United Kingdom; 2 Department of Pharmaceutical Microbiology, Faculty of Pharmacy, Menoufia University, Shebin Elkom, Menoufia, Egypt; 3 Microbiology and Immunology Department, Faculty of Pharmacy, Beni-Suef University, Beni-Suef, Egypt; Bharathidasan University, INDIA

## Abstract

Literature surveys, taxonomical differences, and bioassay results have been utilized in the discovery of new natural products to aid in Actinomycetes isolate-selection. However, no or less investigation have been done on establishing the differences in metabolomic profiles of the isolated microorganisms. The study aims to utilise bioassay- and metabolomics-guided tools that included dereplication study and multivariate analysis of the NMR and mass spectral data of microbial extracts to assist the selection of isolates for scaling-up the production of antimicrobial natural products. A total of 58 actinomycetes were isolated from different soil samples collected from Ihnasia City, Egypt and screened for their antimicrobial activities against indicator strains that included *Bacillus subtilis*, *Escherichia coli*, methicillin-resistant *Staphylococcus aureus* and *Candida albicans*. A number of 25 isolates were found to be active against *B*. subtilis and/or to at least one of the tested indicator strains. Principal component analyses showed chemical uniqueness for four outlying bioactive actinomycetes extracts. In addition, Orthogonal Projections to Latent Structures Discriminant Analysis (OPLS-DA) and dereplication study led us to further select two outlying anti-MRSA active isolates MS.REE.13 and 22 for scale-up work. MS.REE.13 and 22 exhibited zones of inhibition at 19 and 13 mm against MRSA, respectively. A metabolomics-guided approach provided the steer to target the bioactive metabolites (P<0.01) present in a crude extract or fraction even at nanogram levels but it was a challenge that such low-yielding bioactive natural products would be feasible to isolate. Validated to occur only on the active side of OPLS-DA loadings plot, the isolated compounds exhibited medium to weak antibiotic activity with MIC values between 250 and 800 μM. Two new compounds, P_24306 (C_10_H_13_N_2_) and N_12799 (C_18_H_32_O_3_) with MICs of 795 and 432 μM, were afforded from the scale-up of MS.REE. 13 and 22, respectively.

## Introduction

Natural products research has played a vital role in drug discovery and development during the last decades [1]. However, after spans of research and screening of microbial isolates for their natural products production, the chance for discovery of new natural products from microbial origin has declined over the years. Meanwhile, antimicrobial resistance of pathogens is increasingly becoming very severe. Therefore, the urgent need for new antimicrobial agents has augmented the interest of scientists to discover new antibiotics from natural sources [2].

About 45% of the 22,000 reported microbial natural products are produced by actinomycetes, 38% are fungal metabolites and only 17% are from unicellular bacteria [3, 4]. Actinomycetes have been known as a rich source of diverse secondary metabolites with various biological activities [5]. Species of the genus *Streptomyces* have been described to produce about 75% of the reported metabolites from actinomycetes [6, 7]. These secondary metabolites are mainly biosynthesized by either polyketide or non-ribosomal peptide synthetases [8]. Actinomycetes belong to the phylum *Actinobacteria*, characterized by fungal morphology and are filamentous Gram-positive bacteria with high GC (guanine-cytosine) content. Actinomycetes are distributed in many ecosystems including soil where they play an essential role in recycling industrial wastes [8]. Egyptian soils have not been vastly investigated for their actinobacterial reservoir and only few reports have been published on actinobacteria isolated from this ecosystem [9–11].

Conventionally, the process of isolate selection and prioritization for further chemical isolation work depended on either literature surveys, bioactivity screening, or phylogenetic analysis of the isolates [12]. However, the strains of the same genus of bacteria which appear morphologically identical may produce diverse chemical profiles and hence, different biological activities [13, 14]. On the other hand, isolates with vast differences in both their morphology and phylogeny may have a similar metabolome [15]. An excellent strategy to reduce the chance of isolating known compounds is by dereplication of secondary metabolites from each member of the microbial collection prior to selecting the isolates for further chemical isolation work [16]. Therefore, a new drug discovery strategy is using dereplication studies in parallel to multivariate data analysis [17–20]. Dereplication is briefly defined as the quick identification of metabolites detected in a crude extract or fraction on the basis of a database of earlier reported secondary metabolites [17, 21, 22].

Dereplication is considered a critical metabolomic tool used in isolate selection. Metabolomics is a holistic analysis of the small compounds with molecular weight of less than 1000 Da in a biological system at a specific time under a certain set of conditions [23]. LC-HRMS and NMR based metabolomics is an efficient analytical tool to examine mixtures of compounds in a biological system to aid in the discovery of new natural products [15, 24–26]. Statistical approaches like unsupervised and supervised multivariate data analysis have been used to check variation between different sample groups in terms of their chemical shifts in NMR and *m/z* ratio in HRMS [27]. Metabolomic tools have been used to optimize the best fermentation conditions for microbial isolates to yield their bioactive secondary metabolites [17, 21, 22] as well as in the investigation and comparison between intra- and extra-cellular produced secondary metabolites [27, 28].

The main aim of the present study was to strengthen bioassay-guided tools by using a metabolomics approach in prioritizing isolate selection from a collection of Egyptian soil Streptomyces while the concrete objective is to statistically define and pinpoint target novel bioactive metabolites as well as known metabolites with new bioactivity directly from the crude bacterial extracts prior to accomplishing a tedious chemical isolation and structure elucidation work [17, 19]. In this paper, we would like to present a dereplication methodology utilizing multivariate analyses to compare and identify the interesting bioactive secondary metabolites directly from crude extracts of bacterial isolates. As demonstrated by our earlier works on other natural resources [29–37], such procedures will assist in optimizing the scale-up and chromatographic isolation work on this yet underexplored collection of actinomycetes from Egyptian soil isolates [9]. Anti-MRSA bioactivity was the targeted activity in this study, which directed the prioritization of isolates for further chemical work. Although many previous studies on actinobacteria from Egypt have been published [38–44], most of these studies conducted a bioassay-guided approach for screening the active isolates without any consideration for chemical dereplication and thus, a metabolomics-based investigation is the core of our study.

## Materials and methods

### Samples collection and actinomycetes isolation

Several soil samples were collected between 2014 and 2016 from Ihnasia City located in Beni-Suef Governate, Egypt. The ancient city of Ihnasia is located approximately 15 km west of the modern city of Beni-Suef. Collection were done during the different seasons namely spring when the weather is cooler and dry (March to May), summer that is hot and very dry (June to August), and in autumn that is warm and a bit more humid (September to November) (S4 Table). No permits were required for site access to collect soil samples for academic research work in Egyptian institutions for higher education. The soil samples were collected from a depth of 15 to 20 cm of the superficial layers of the soil into sterile bags. Actinobacterial cultivation, isolation, and purification were done using a soil dilution plate technique [45] in International Streptomyces Project 4 (ISP4) medium. ISP4 medium was supplemented with rifampicin (10 μg/ml) to inhibit the growth of the fast-growing Gram-negative bacteria and nystatin (50 μg/ml) to inhibit fungal growth and contamination. One gram of each soil sample was diluted with 9 ml of 0.9% saline, then mixed, homogenized and prepared to ten-fold serial dilutions up to 10^−4^. One ml from dilutions (10^−2^, 10^−3^ and 10^−4^) for each soil sample was plated out on ISP4 agar plates using sterile cotton swabs, and the plates were incubated for 7 d at 30°C. Actinomycetes-like colonies depending on their morphological characters, pigment diffusion, and coloration of their mycelia [46, 47] were picked and streaked several times on ISP4 agar plates until pure actinobacterial colonies were isolated. The isolated actinomycetes were maintained on agar plates for short-term storage and for long-term storage, the isolates were archived by storing in 30% glycerol in ISP4 broth at -80°C.

### Antimicrobial activity screening

*In vitro* screening for antimicrobial activity of the actinobacterial extracts was done by cup diffusion method against four different indicator strains [48]. The indicator strains used were *B*. *subtilis* (ATCC 21228), *Escherichia coli* (*E*. *coli*) (ATCC 25922), MRSA (ATCC 43300) and *Candida albicans* (ATCC 24433). Bacterial extracts were prepared by extraction of the extracellular metabolites with ethyl acetate from 500 ml of initial broth cultures of ISP4 previously seeded with 5% bacterial inoculum in Tryptone soya broth (TSB) and incubated at 30°C for 7 d on a rotary shaker incubator at 160 rpm. Briefly, after surface inoculation of the tryptone soya agar plates with 100 μl of the standard strains, a sterile borer was used to form 4 cups in each plate with a diameter of 10 mm, then 150 μl of each extract solution (1 mg/ml) was added in the cups. Finally, after incubation of the plates for 24 h, the antimicrobial activities were assessed from the inhibition zones around the cups. Streptomycin solution in concentration of 1 mg/ml was used as the reference standard or positive control.

### Bacterial fermentation and extraction

All bioactive isolates were selected for further metabolomic profiling studies. Fermentation of the selected bioactive isolates was done by sub-culturing them in TSB and incubated at 30°C for 3 d. Twenty-five milliliters of the TSB (5% inoculum) was used to inoculate 500 ml of ISP4 broth in 1 L flask and then incubated at 30°C for 7 d on rotary shaker incubator at 180 rpm. Culture growth was terminated with 1:1 v/v HPLC grade ethyl acetate and left for 24 h, then filtered using a Buchner funnel with 100 mm filter papers. The filtrate was then transferred to a 1 L separating funnel and liquid–liquid partitioning was accomplished between water and ethyl acetate. The aqueous filtrate was partitioned three times with equal volumes of ethyl acetate to extract all organic metabolites. Ethyl acetate fractions were concentrated under *vacuo* with a rotary evaporator. The dried crude extract was weighed and reconstituted with the appropriate solvent for chemical analysis and bioassay screening [49, 50].

The culture conditions and extraction procedures for the scale-up of the selected isolates (MS.REE. 13 and MS.REE. 22) were the same as that of the small-scale fermentation of the bioactive isolates using ISP4 broth as mentioned above. In the present study, ISP4 broth was seeded by 5% tryptone soya broth which contains pancreatic digest of casein, dextrose and enzymatic digest of soya bean.

### Mass spectrometry

Aliquots of crude bacterial extracts along with medium (ISP4) control samples were prepared at 1 mg/ml solutions in methanol. Methanol solvent blank was also included in the analysis. LC-HRMS of the samples was run on an Accela HPLC (Thermo Scientific, Germany) coupled to an Exactive mass spectrometer (Orbitrap, Germany) with an electrospray ionization source according to [31]. Briefly, mass accuracy of the mass detector was set to less than 3.0 ppm. The instrument was calibrated internally using lock masses and externally according to the instructions of the manufacturers. High resolution mass spectrometry was carried out over a mass range of 150–1500 *m/z* in both negative and positive ionization switch modes. The instrument capillary temperature was set at 270°C with a spray voltage of 4.5 kV. The LC-HRMS experiments were carried out with ACE5 Excel 3 Super C18 column (5 μm × 150 mm × 3 mm) (Hichrom Limited, Reading, UK), using 10 μl as injection volume from each vial. The two mobile phases used to carry out the mass spectrometry were ultrapure water with 0.1% formic acid (Solvent A) and HPLC grade acetonitrile with 0.1% formic acid (Solvent B). The gradient started with 10% solvent B at a flow rate of 0.3 ml/min and the percentage of solvent B was linearly increasing to 100% within 30 minutes. Then remained isocratic at 100% B for 5 minutes before decreasing again within one minute to 10% B which continued isocratic at 10% B as final washing for 10 minutes before the next sample injection. The sample tray and the column oven temperatures were maintained at 4°C and 20°C respectively. The run sequence started with the blank solvent then the blank medium followed by the samples. The HRMS data of the blanks and the samples were acquired using Xcalibur software version 3.

### NMR spectroscopy

For NMR study, NMR samples for both culture medium and bacterial extracts were prepared by dissolving 5 mg of the crude organic extracts of the medium and bacterial isolates in 600 μl DMSO-*d*_6_ then transferred to 5 mm 7″ NMR tubes [19]. Correlation Spectroscopy (COSY) and proton NMR was carried out on a 400 MHz Jeol-LA400 FT-NMR spectrometer system equipped with a 40TH5AT/FG probe. For both COSY and proton NMR experiments, 16 scans were recorded. Proton and COSY spectra were processed using MestReNova (Mnova 10.00, Mestrelab Research SL, US) software. For Proton Spectra, smoothing with Whittaker Smoother, baseline correction with Whittaker Smoother, apodization with Gaussian 1.00 and manual phase correction were carried out in MestReNova (Mnova 10.00). For COSY analysis, smoothing with Whittaker Smoother, reducing t1 noise and symmetrizing as COSY-like were done, then spectra from the bacterial extracts were overlaid with the ISP4 medium spectrum (control) to differentiate correlations from metabolites produced by the bacteria from those coming from the culture medium.

### Metabolomic profiling analysis of the LC-HRMS and NMR data

Using MassConvert tool from Proteowizard, the raw HRMS data were split into two different data sets (positive mode data set and negative mode data set) [51]. The split data set in the format of mzML was imported into MZmine 2.20 [52] to extract features from the raw data. Data processing was carried out for the individual data sets using MZmine 2.20 following a multi-step protocol [31], commencing with peak detection (mass detection and chromatographic builder) followed by deconvolution, deisotoping, filtering, alignment and gap filling. Finally, adducts and complexes were identified while respective ion peaks were eliminated to minimize the mis-assignment of features and to predict molecular formulae using an accuracy of 5 ppm for each feature were carried out.

The generated CSV files for positive and negative modes by Mzmine 2.20, were copied into an in-house Excel macro for further data clean-up [19]. Briefly, an in-house Excel macro was used to subtract background ion peaks found in the solvent blank and medium, then simultaneously both negative and positive ionization modes data files generated by MZmine were combined in preparation for multivariate data analysis and finally to dereplicate the metabolites in the samples with those in the Dictionary of Natural Products (DNP) database. All peaks of the solvent blank, which had peak intensity greater than 1 × 10^4^ were subtracted manually from the samples’ peaks. Peaks originating from the ISP4 medium were also subtracted from the samples by applying an algorithm, which calculates each *m/z* intensity in both medium and bacterial extracts. All ion peaks coming from the medium were subtracted leaving only those features with peak intensity 20 times greater in the samples than in the ISP4 medium. The data sets of both negative and positive ionization modes data sets were combined by macro after removing the effect of the medium enabling ion peaks that were observed in both modes and one of them to be overlaid for further statistical analysis. Finally, an Excel macro was used for dereplication of each *m/z* ion peak with metabolites in the customized database (DNP). This database provided us with the details on the already known compounds in the bacterial extracts and the unknown hits which can help for targeting new bioactive metabolites in the extracts and help in selecting the promising isolates for further scale-up as well.

The SIMCA data sheet generated by the Excel macro was then exported to SIMCA 15.0.1 (Umetrics, Umeå, Sweden) to do the multivariate data analysis (MVA). The data set was analyzed using both PCA and Orthogonal Projections to Latent Structures Discriminant Analysis (OPLS-DA), respectively an unsupervised and a supervised MVA method with Pareto scaling [53]. PCA was used to compare the chemical profiles of the different samples without classifying them while OPLS-DA was generated by comparing the chemical profiles by pre-grouping the isolates according to their bioassay screening results against MRSA and *E*. *coli*. The metabolomics profile tables of interesting bioactive metabolites were deduced from the S-plots of the OPLS-DA by selecting the discriminating compounds found on the bioactive extracts and comparing them to the dereplication data sheet generated by the Excel macro. Depending on the results of the dereplication study, bioassay screening, and the multivariate data analysis of the bioactive bacterial extracts, promising isolates were selected for further scale-up.

For proton NMR spectra, the processed spectra of the medium and different extracts were stacked using MestReNova software (Mnova 10.00) and using an advanced PCA tool of Mnova 10.00, a spectral data set was generated for further data analysis. The data set between 0.5 to 12.5 ppm of the medium and extracts were copied to an Excel sheet and the peaks of the solvent used (DMSO and water peaks) were deleted. Finally, the resulting peak list was exported to SIMCA 15.0.1 for multivariate analysis (PCA and OPLS-DA).

### Taxonomical identification of the selected isolates through molecular biology

DNA of two selected isolates for the scale-up was extracted. A single colony of each isolate from the agar plate was inoculated to 4 ml of ISP2 broth and incubated at 30°C for 3 d. One ml of the bacterial broth culture was centrifuged for 10 min at 3000 rpm to separate the cells. The cells were suspended in 300 μl of Tris-EDTA buffer with 5 mg/ml lysozyme and 0.1 mg/ml RNase, vortexed and incubated at 37°C for 30 min to confirm that the cells were properly lysed. Then, 50 μl of 10% SDS and 85 μl of 5 M NaCl were added and mixed thoroughly. To the suspended lysed cells, 400 μL of phenol: chloroform: isoamyl alcohol (25:24:1) were added, vortexed for 30 s and centrifuged at 13200 rpm and the top aqueous phase was transferred to a new tube. A 400 μl solution of chloroform: isoamyl alcohol (24:1) was added, vortexed for 30 s and centrifuged for 10 min at 13200 rpm and the aqueous layer was transferred to new tube. A volume of 500 μl isopropanol was added to the aqueous layer, mixed by inversion and let sit at room temperature for 5 min. Finally, spin down at 10000 rpm for 5 min to pellet the DNA, remove isopropanol very gently and wash out the DNA with 1 ml cold 70% ethanol. The ethanol was gently removed. The final DNA pellet was left to dry on bench for 1 h and then resuspended in 50 μl sterile H_2_O and kept in the fridge at 4°C for further work.

For taxonomical identification of the selected isolates through molecular biology, 16S rRNA genes of both isolates were amplified by PCR using a universal 27F primer (5`-AGAGTTTGATCMTGGCTCAG-3`) and universal 1492R primer (5`-GGTTACCTTGTTACGACTT-3`) [54]. The PCR amplification was carried out in total volume of 25 μl reaction mixture which consisted of 1 μl genomic DNA, 0.25 μl of each primer, 0.2 μl MyTaq DNA polymerase (Bioline), 5 μl of 5x reaction buffer (New England Biolabs) to which dNTPs were added and finally sterile water was added to make the total reaction volume 25 μl. The PCR was carried out according to the following thermal protocol: initial denaturation for 5 min at 95°C, followed by 29 cycles starting with denaturation at 95°C for 10 s, then annealing at 55°C for 30 s and extension for 3 min at 72°C with final extension after finishing the cycles at 72°C for 4 min. Agarose gel electrophoresis was used to check the PCR amplification by loading 5 μl of each PCR product in 1% (w/v) agarose at 100 V for 60 min and compared with 1 Kb ladder (Promega) as reference using GelRed for staining (Biotium). Then, the PCR products were purified using ISOLATE II PCR and Gel Kit (Bioline) following the manufacturer’s protocol for PCR products purification. Finally, a concentration of 10 ng/μl for each purified PCR product was prepared and sent to GATC (Germany) for sequencing using the 27F and 1492 R primers.

Good quality sequences were selected and compared to the GenBank database using the Megablast tool of National Center for Biotechnology Information (NCBI) to optimize contrasting the amplified sequences against only the highly similar sequences in the GenBank database to identify the closest related strains to our amplified sequences [55]. Multiple sequence alignment and phylogenetic analysis of the isolates sequences were carried out using MEGA7 software [56].

## Results and discussion

### Actinomycetes isolation and antimicrobial activity screening

A total of 58 bacterial strains were isolated and purified from soil samples from Egypt by several successive and repetitive inoculation. Many previous reports confirmed the richness of soil niches with important actinomycete isolates [57, 58]. Isolates were characterized as actinomycetes depending on their morphological characters, pigment diffusion, and coloration of their mycelia.

ISP4 broth was the chosen culture media as it exhibited consistency in the antimicrobial activity for all bioactive strains, which as well indicated stability of the type of bioactive metabolites between cultivation and scale-up during the present study. As mentioned in a previous report, polysaccharides like starch as carbon source present in ISP4 is commonly used for optimum production of antibiotics due to supporting slower microbial, growth which is suitable for antibiotics biosynthesis [59]. Also, as shown in many previous studies in the literature, the use of ISP4 in both isolation and fermentation work has yielded good results [9, 60–63]. ISP4 was the best culture medium for isolating actinomycetes when compared to other media such as ISP2, ISP3, actinomycete isolation agar, glucose asparagine agar and starch-yeast extract agar [60]. ISP4 as a culture medium also exhibited suitable production of antimicrobial agents by Streptomyces in agar plates. Extracellular metabolomic screening of fermentation cultures of different Streptomyces isolates in modified ISP broth medium (with added 0.4% yeast extract) yielded various analogues of pyrromycin, erythromycin, doxorubicinol, and some other putative novel metabolites indicating that ISP4 broth was most appropriate for the production of various classes of antibiotics [61]. Interestingly, another earlier study showed that ISP4 broth with 2.5% starch instead of 1% was the best media for production of antibiotics by a Streptomyces strain isolated from soil samples collected from Egypt as well [62], as ISP4 broth was compared to other culture media such as ISP2 broth, tryptone-yeast extract-glucose broth, starch-nitrate broth medium in this particular study. More recently, ISP4 was also used to culture Streptomyces strains collected from Egyptian soil isolates for producing various classes of bioactive compounds both as antibacterial and anticancer agents [9]. ISP4 broth was also used for the discovery and overproduction of the nucleoside antibiotic A201A from marine-derived actinomycete *Marinactinospora thermotolerans* [63].

Extracts of collected bacterial isolates were initially screened against *B*. *subtilis* in various solvents using hexane, chloroform, dichloromethane and ethyl acetate by cup diffusion method [48] (S1 Fig). Hexane and chloroform extracts did not exhibit any antimicrobial activity in comparison to ethyl acetate and dichloromethane. A second screening for antimicrobial activity against four indicator strains (*B*. *subtilis*, *E*. *coli*, MRSA and *C*. *albicans*) was repeated with ethyl acetate extracts of the bacterial isolates. Ethyl acetate was the chosen extracting solvent because it was able to isolate most of the bioactive semi-polar components while efficient partitioning of inactive long alkyl chains and sugar molecules could be accomplished. Ethyl acetate was also considered to be less expensive and less toxic to handle than dichloromethane. Positive inhibition of at least one of the tested indicator strains was exhibited by 25 extracts (43% of the collected isolates) as shown in Table 1. A clear zone of inhibition of greater than 12 mm diameter was considered as active. In many previous reports, a high percentage of biological activity has been exhibited by soil actinomycete isolates [9, 64, 65], which matches our antimicrobial activity screening results. A broad spectrum of antibacterial activity against all the tested bacteria (*B*. *subtilis*, *E*. *coli* and MRSA) was demonstrated by five isolates as earlier reported for a collection of soil actinomycete isolates from Egypt [9].

**Table 1:** Antimicrobial activity of the actinomycetes isolates against different indicator strains used in the study. The zones of inhibition were measured and recorded as active when it was greater than 12 mm. Twenty-five bioactive isolates were found and presented in this table out of the total 58 isolated actinomycetes.

Isolate Extract	vs *B*. *subtilis*	vs MRSA	vs *E*. *coli*	vs *Candida albicans*
**MS.REE. 1**	Active	Active	Inactive	Inactive
**MS.REE. 3**	Active	Inactive	Active	Inactive
**MS.REE. 4**	Active	Active	Inactive	Inactive
**MS.REE. 6**	Active	Inactive	Active	Inactive
**MS.REE. 9**	Active	Inactive	Inactive	Inactive
**MS.REE. 10**	Active	Active	Active	Inactive
**MS.REE. 12**	Active	Inactive	Inactive	Inactive
**MS.REE. 13**	Active	Active	Active	Inactive
**MS.REE. 14**	Active	Inactive	Active	Inactive
**MS.REE. 16**	Active	Inactive	Active	Inactive
**MS.REE. 17**	Active	Inactive	Inactive	Inactive
**MS.REE. 18**	Active	Inactive	Inactive	Inactive
**MS.REE. 19**	Active	Inactive	Active	Inactive
**MS.REE. 22**	Active	Active	Active	Inactive
**MS.REE. 24**	Active	Inactive	Inactive	Inactive
**MS.REE. 25**	Active	Active	Active	Inactive
**MS.REE. 26**	Active	Active	Active	Inactive
**MS.REE. 27**	Active	Active	Inactive	Inactive
**MS.REE. 28**	Active	Active	Inactive	Inactive
**MS.REE. 29**	Active	Inactive	Active	Inactive
**MS.REE. 32**	Active	Inactive	Inactive	Inactive
**MS.REE. 33**	Active	Inactive	Inactive	Inactive
**MS.REE. 34**	Active	Inactive	Inactive	Inactive
**MS.REE. 36**	Active	Inactive	Active	Inactive
**MS.REE. 46**	Active	Inactive	Inactive	Inactive

### Data processing and clean-up

**Fig 1:**
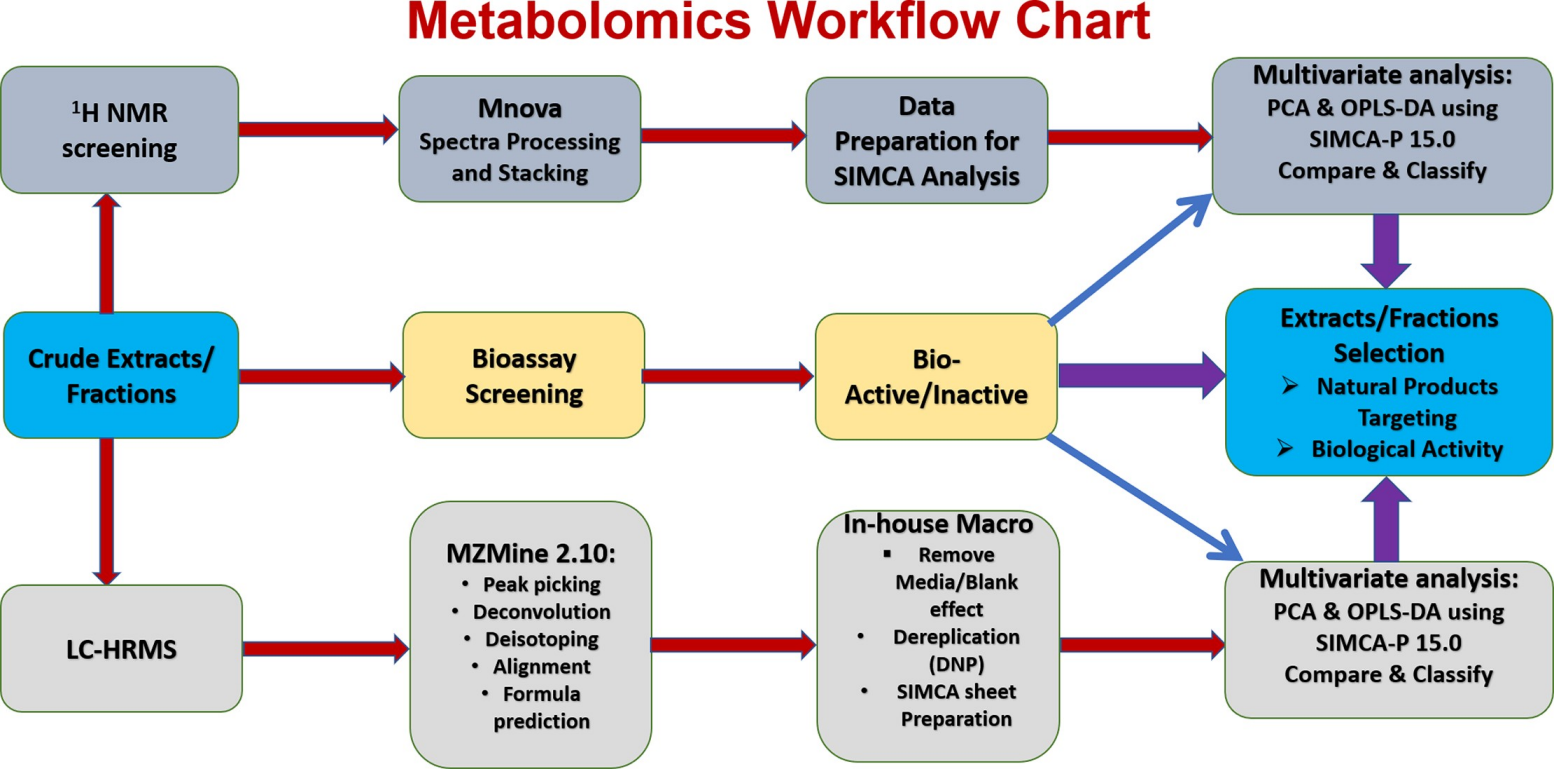
Metabolomics workflow chart to aid isolates selection.

Metabolomic profiles of the bioactive crude extracts were investigated using NMR and LC-HRMS according to the workflow chart shown in Fig 1. After processing the HRMS data using MZmine 2.20 as described in the methodology section, data clean-up was done by importing the CSV files of both positive and negative modes data to an Excel macro. The Excel macro helped in overcoming the limitations of MZmine 2.20 by combining the data of positive and negative modes, which is particularly important for metabolites ionizing on both modes that enabled merging the features observed in either or both modes together. Thus, this valuable Excel macro optimized the processing of each ion peak along with those detected only in one mode. This is also essential for data analysis of compounds like phenolics and anthraquinones, which ionize only in the negative mode and are poorly ionize in the positive mode [22].

Another problem for data analysis of bacterial extracts is the presence of background peaks from the culture media. Most of the constituents of the culture media should be utilized by the bacterial isolates during the fermentation process. However, some of the media constituents may remain unutilized while trace amounts can be detected by LC-HRMS. These interfering peaks from the medium could be misleading during the dereplication study, so the medium was instead considered as one of the blank samples. Then, medium interfering peaks were removed using the in-house Excel macro as mentioned in the methodology section.

### Metabolomic profiling analysis for isolate prioritization

**Fig 2:**
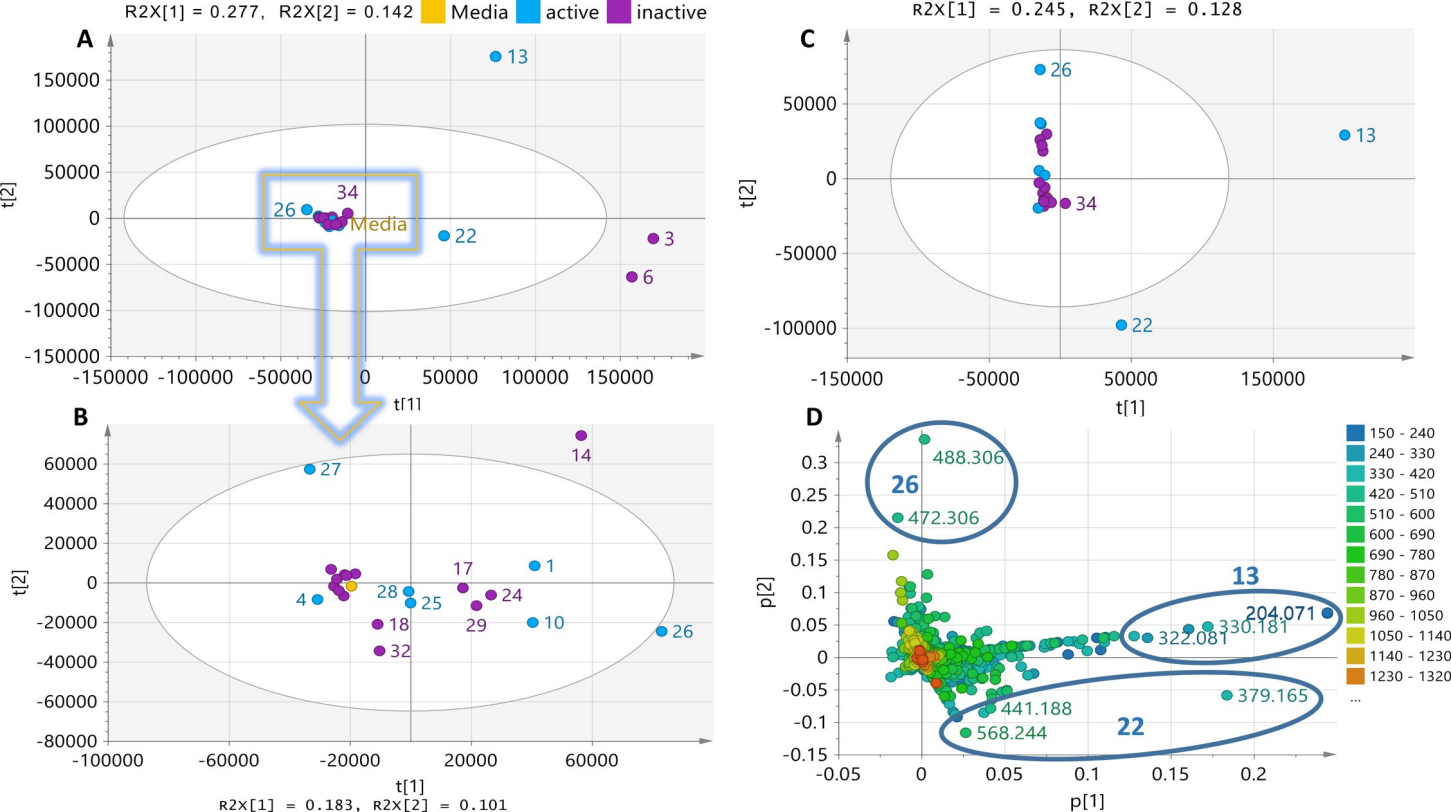
PCA of LC-HRMS data of 25 bacterial extracts tested against MRSA. (**2A)** Scores plot of the 25 extracts. (**2B)** Scores plot of 21 extracts after removal of outlying samples MS.REE. 3, 6, 13, and 22. (**2C) Sc**ores plot after removal of the outlying inactive isolates MS.REE. 3 and 6. (**2D)** Corresponding loadings plot of ion peaks in *m/z* highlighting the significant metabolites of the outlying bioactive isolates MS.REE. 13 and 22.

PCA analysis of the 25 extracts for three components gave R^2^ (goodness of fit) and Q^2^ (predictability) values at 0.5 and 0.1, respectively, where 84% of the isolates clustered together. The projected R^2^ and Q^2^ values are highly application dependent [53]. Biological PCA models are expected to yield R^2^ and Q^2^ values of at least 0.5 and 0.4, respectively. In general, R^2^ should not exceed Q^2^ by more than 2 units. The low predictability of the model was due to the low difference in chemical profiles of 84% of the analyzed samples, which consisted of 21 isolates. Isolates MS.REE. 3, 6, and 13 were classified as outliers within the tolerance ellipse of 95% hotelling at T2 as shown in the scores plot (Fig 2A). Outlying isolates indicated chemical uniqueness as they lay far from the other isolates clustered in the center or within the eclipse. Conversely, 84% of the isolates clustered with the media control except for MS.REE-22. However, this clustering of 84% of the isolates was not because of their low chemical diversity but would be due to the relatively vast difference in profiles of these extracts when compared to the outliers in terms of their yield and complexity. This was evident on the percentage variation on PC1 and PC2 at 27.7 and 14.2, respectively before excluding the outliers, which was then subsequently reduced to 18.3 and 10.1, respectively. The higher percentage variation in the presence of the outliers merely implied that 84% of the isolates have a lower capability of affording the same intensity of variation of secondary metabolites in comparison to the outliers. Additionally, the chemical diversity of 84% of the tested isolates was validated by extracting the PCA scores plot of the cluster from the four outlying extracts MS.REE. 3, 6, 12, and 22 (Fig 2B). As shown in Fig 2B, 67% of the extracts segregated from the medium with no clear clustering, particularly those active samples against MRSA, indicating their distinct chemical profiles although they were less diverse than the outliers. This further suggested that ISP4 was indeed an appropriate standard media for this bioscreening campaign for this selection of Actinomycetes. Outliers MS.REE. 3 and 6, which were only found active against *E*. *coli* and inactive against MRSA, were then excluded for further multivariate analysis (Fig 2C). In this case, this was the first criteria to consider MS.REE.13 and 22 for scale-up work. MS.REE.13 exhibited zones of inhibition at 19 and 18 mm against MRSA and *E*. *coli*, respectively, MS.REE.22 gave 13 and 18 mm, respectively. MS.REE. 3 and 6 yielded inhibition zones of 18 and 22 mm against *E*. *coli*, respectively. Streptomycin was used as positive control and displayed a 25 mm zone of inhibition against both MRSA and *E*. *coli*.

The loadings plot of the *m/z* ion peaks shown in Fig 2D indicated the metabolites of the isolates, matching the same quadrant position of the isolates in the scores plot (Fig 2C). As shown in the loadings plot (Fig 2D), the metabolites responsible for the separation of isolate MS.REE.13 had lower molecular weights ranging from 200 to 400 Da, while MS.REE. 22 outlier metabolites had higher molecular weights ranging from 300 to 500 Da. Both MS.REE. 13 and 22 showed promising activity against MRSA, *E*. *coli* and *B*. *subtilis* indicator strains (Table 1), while MS.REE. 3 and 6 showed no activity against MRSA, which was the main target activity for initial scale-up work.

**Fig 3:**
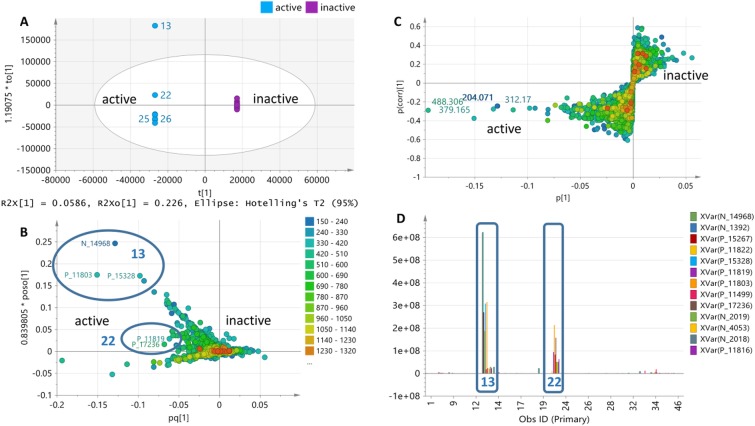
OPLS-DA of LC-HRMS data of 23 extracts tested against MRSA after removal of the outlying inactive isolates MS.REE. 3 and 6. (**3A**) Scores and (**3B**) loadings plot of ion peaks in *m/z*. (**3C**) Loadings S-plot against MRSA with predicted bioactive metabolites labelled with their *m/z* ion peaks on the left lower quadrant. (**3D**) Column plot indicating the relative concentration in ion peak intensity of predicted bioactive metabolites found in the bioactive isolates MS.REE. 13 and 22. The predicted bioactive metabolites are listed under their MZmine IDs in Table 2 with their P values as determined from the S-plot.

**Fig 4:**
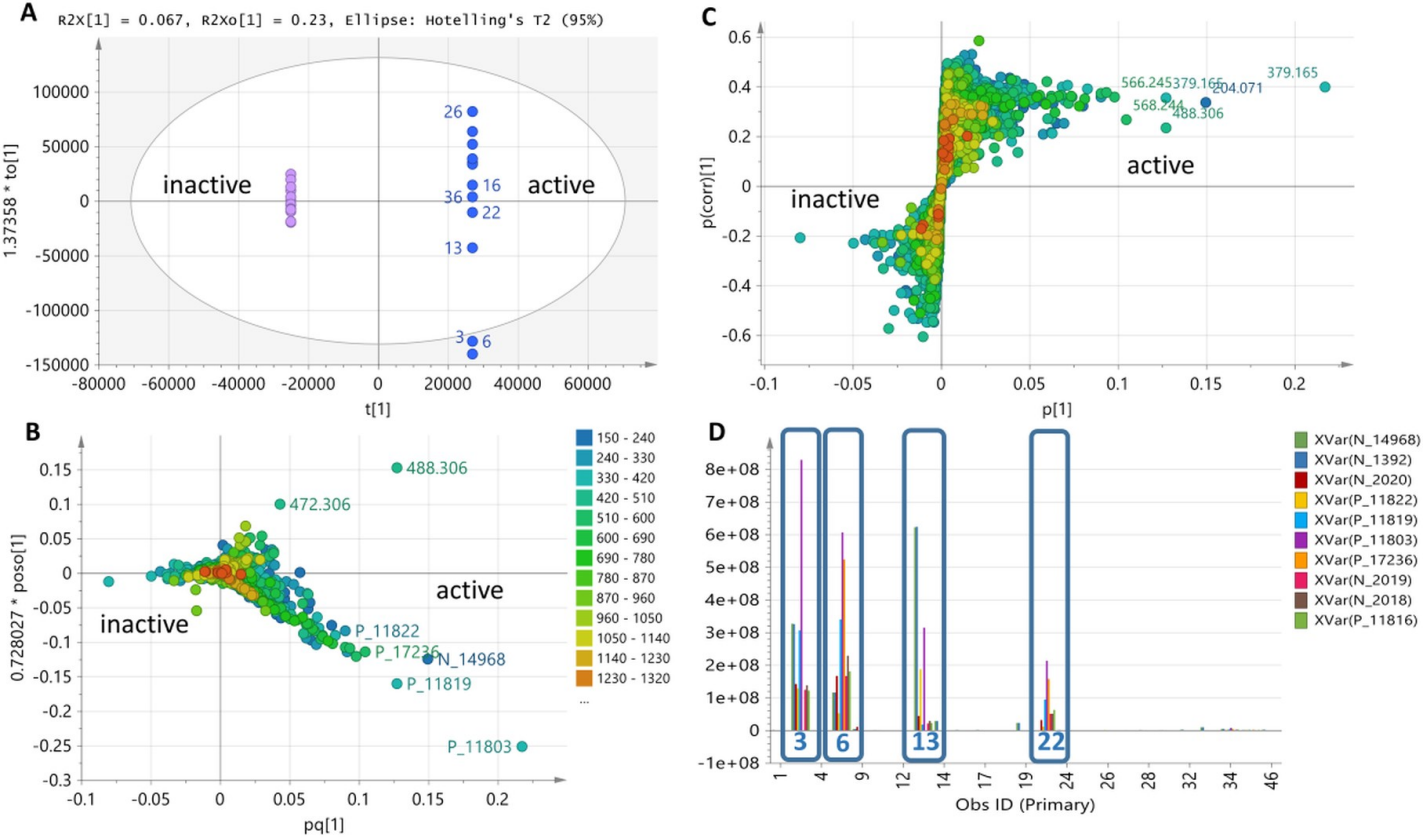
OPLS-DA of LC-HRMS data of 25 extracts tested against *E*. *coli*. (**4A**) Scores and (**4B**) loadings plot of ion peaks in *m/z*. (**4C**) Loadings S-plot against *E*. *coli* with predicted bioactive metabolites labelled with their *m/z* ion peaks on the left lower quadrant. (**4D**) Column plot indicating the relative concentration in ion peak intensity of predicted bioactive metabolites found in the bioactive isolates MS.REE. 3, 6, 13, and 22. The predicted bioactive metabolites are listed under their MZmine IDs in Table 2 with their P values as determined from the S-plot.

The antimicrobial activity of the isolates was used to generate the supervised model (OPLS-DA) by classifying the isolates according to their bioassay screening results against MRSA and *E*. *coli* (Figs 3 and 4). The OPLS-DA model based on the bioactivity of the 23 isolates against MRSA (Fig 3A) generated from 19 orthogonal X components for MRSA bioactivity gave an R^2^ value of 0.99 but with poor predictability Q^2^ value of 0.07. Percentage variation between groups was 5.86% while within groups was 22.6%. The higher percentage variation within groups can be explained by the separation of the outlying bioactive isolate MS.REE.13. However, for visualization purposes, it was possible to define the bioactive target metabolites responsible for separating MS.REE. 13 and 22 not only from the inactive isolates but as well as from the rest of the bioactive isolates as shown on the OPLS-DA loadings plot (Fig 3B). The active and inactive end of the S-plot matches the position of the respective groups as found on the OPLS-DA scores plot is either positioned on the left or right quadrant of the ellipse. The detected bioactive secondary metabolites were dereplicated and listed on Table 2. The loadings S-plot shown on Fig 3C indicated the bioactive metabolites separating the active from those of the inactive isolates against MRSA. The bioactive discriminating metabolites against MRSA were listed on Table 2. Smaller P values ≤ 0.10 indicated stronger evidence for predicted bioactivity against MRSA, most of which were detected from isolates MS.REE. 13 and 22. The relative abundance of these discriminating active metabolites was shown on Fig 3D. These included ion peaks at *m/z* 379.165 [M+H]^+^ and 566.245 [M-H]^-^ with their predicted molecular formulas C_22_H_22_N_2_O_4_ (ring-plus-double-bond equivalent (RDB) = 13) and C_37_H_33_N3O3 (RDB = 23), respectively.

**Table 2:** Predicted discriminating metabolites found against MRSA and *E*. *coli* determined from their respective OPLS-DA S plots as shown in Figs 3 and 4. Ionization mode is designated on MZmine ID as P for positive mode [M+H]^+^ and N for negative mode [M-H]^-^. Highlighted rows list the unique metabolites found for the respective bioactivities. Smaller P values ≤ 0.10 indicate stronger evidence for predicted bioactivity.

MZmine ID	RT (Min)	m/z	MW (accuracy in ppm)	Formula Prediction / RDB	Hits in DNP database (compound no) / Source	Isolate no. w/ highest intensity	P value MRSA bioactivity	P value *E*. *coli* bioactivity
P_11499*	5.94	441.188	440.181 (-0.42)	C_21_H_24_N_6_O_5_ RDB = 13	(E)-*N*^2^-isobutyryl-8-[2-(pyrid-4-yl)-ethenyl]-2’-deoxyguanosine*** (**1**)	22	0.223	inactive end
			440.180 (2.53)	C_20_H_28_N_2_O_9_ RDB = 8	*N*^α^-(benzyloxycar bonyl)-N^δ^-(β-D-fucopyranosyl)-L -glutamine O-methyl ester*** (**2**)			
**N_14968**	5.97	204.071	205.079	no prediction	no hits	13	0.255	0.099
**P_11822**	8.19	322.081	321.075 (-2.53)	C_17_H_11_N_3_O_4_ RDB = 14	*N*”-(5-hydroxy-2-oxoindolin-3-ylidene)benzofuran-2- carbohydrazide*** (**3**)	13	0.193	0.080
P_11803*	10.66	379.165	378.158 (-0.98)	C_22_H_22_N_2_O_4_ RDB = 13	2-[3-hydroxy-2-methoxy-1-(1*H*-indol-3-yl) propyl]-1*H*-indole-3-acetic acid** (**4C**)/marine-derived *Rubrobacter radiotolerans*	13	0.078	0.048
**N_4053**	11.35	566.245	567.252 (-0.008)	C_37_H_33_N_3_O_3_ RDB = 23	2,3,6-triphenyl-1-phenylcarbamoyl-4-phenylcarbamoyl hydroxypiperidine*** (**5**)	22	0.082	inactive end
**N_2018**	11.83	566.245	567.252 (-0.008)	C_37_H_33_N_3_O_3_ RDB = 23		22	0.087	0.079
**P_11816**	11.85	590.226	589.221 (-3.03)	C_35_H_31_N_3_O_6_ RDB = 22	4-amino-6,10-diaroyl-7,9-diaryl-8-oxa-2,3-diazaspiro[4,5]deca-3-en-1-ones*** (**6**)	22	0.115	0.064
**N_2020**	12.61	305.138	306.144 (3.18)	C_13_H_18_N_6_O_3_ RDB = 8	*N*’,*N*”-[(2-oxo-1*H*-benzimidazole-1,3(2*H*) -diyl)bis (methylene)] diacetohydrazide*** (**7**)	6	deleted inactive outlier	0.070
**P_11819**	12.68	379.165	378.158 (-0.98)	C_22_H_22_N_2_O_4_ RDB = 13	nocazine A** (**4A**) / marine-derived *Nocardiopsis dassonvillei* HR10-5	13	0.078	0.081
					2'-deoxy clavulanic acid**(**4B**) / *Streptomyces clavuligerus*			
**N_2019**	12.85	566.245	567.252 (-0.35)	C_37_H_33_N_3_O_3_ RDB = 23	2,3,6-triphenyl-1-phenylcarbamoyl-4-phenylcarbamoyl hydroxypiperidine*** (**5**)	22	0.082	0.066
**P_17236**	12.87	568.244	567.237 (0.08)	C_33_H_33_N_3_O_6_ RDB = 19	3’,6’-dimethoxy-N-(4’-((1-methyl piperidin-4-yl)oxy)-3’-nitro-[1,1’-biphenyl]-4-yl-[1,1’-biphenyl]-3-caboxamide*** (**8**)	22	0.230	0.197
P_15328*	14.66	330.181	329.174 (-1.17)	C_18_H_23_N_3_O_3_ RDB = 9	3-(2-(amino) acetamido)benzofuran-2-carboxamide*** (**9**)	13	0.217	inactive end
P_15267*	15.16	312.170	311.163 (-0.03)	C_18_H_21_N_3_O_2_ RDB = 10	talathermophilin E (**10**) / *Talaromyces thermophilus* YM3-4	13	0.211	inactive end

*major peaks found on the base peak chromatogram as presented in S2 Table and S3 Table

**interchangeable

***Chemspider hits from reported antimicrobial synthesized compounds

For the MVA of the bioactivity of 25 extracts against *E*. *coli*, using two components, an R^2^ value of 0.79 was attained. Percentage variation between groups was 6.7% while within groups (also known as internodal variation) was 23%. The separation of the outlying bioactive isolates MS.REE.3 and 6 resulted to the higher percentage variation within groups (Fig 4A). As also shown in Fig 4A, the OPLS-DA scores plot (Fig 4A) for the bioactivity of the isolates against *E*. *coli* revealed the uniqueness of MS.REE. 3 and 6 with metabolites with molecular weights ranging from 200 to 600 Da (Fig 4B). S-loadings plot was also generated for anti-*E*. *coli* bioactivity (Fig 4C). The interesting bioactive secondary metabolites were detected mainly from isolates MS.REE.3, 6, 13, and 22 as shown on Fig 4D designating their relative abundance, which will be targeted to optimize their yield during the scale-up work.

**Fig 5:**
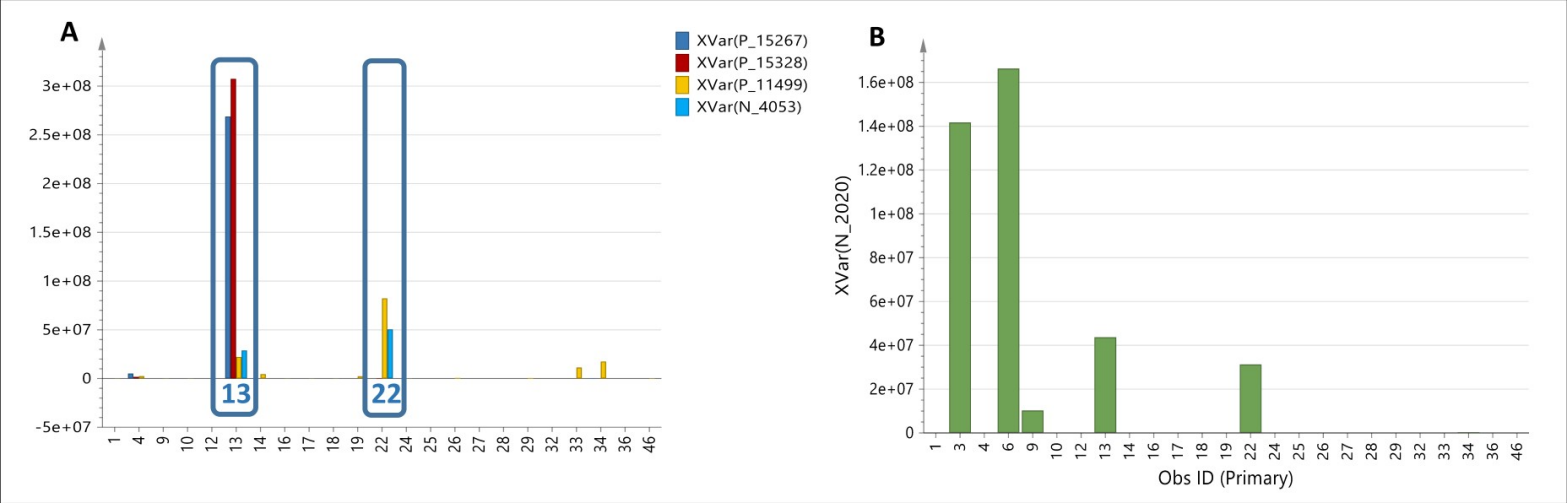
Column plots of the relative abundance of predicted bioactive metabolites against MRSA. (**5A**) and *E*. *coli* (**5B**) unique to isolates MS.REE. 13, 22, 3, and 6, respectively. The predicted bioactive metabolites are listed under their MZmine IDs in Table 2.

Regarding the specificity of the discriminating bioactive metabolites against MRSA and *E*. *coli*, unique metabolites can be assigned for the respective test strains, Table 2 (Fig 5A and 5B). Metabolites anticipated to be specific against MRSA included P_15627, P_15328, P_11499, and N_4053 at *m/z* 312.170 [M+H]^+^, 330.181 [M+H]^+^, 441.188 [M+H]^+^, and 566.450 [M-H]^-^, respectively. On the other hand, N_2020, an ion peak at *m/z* 305.138 [M-H]^-^ was predicted to be specific against *E*. *coli*. Dereplication results are further presented and discussed below.

**Fig 6:**
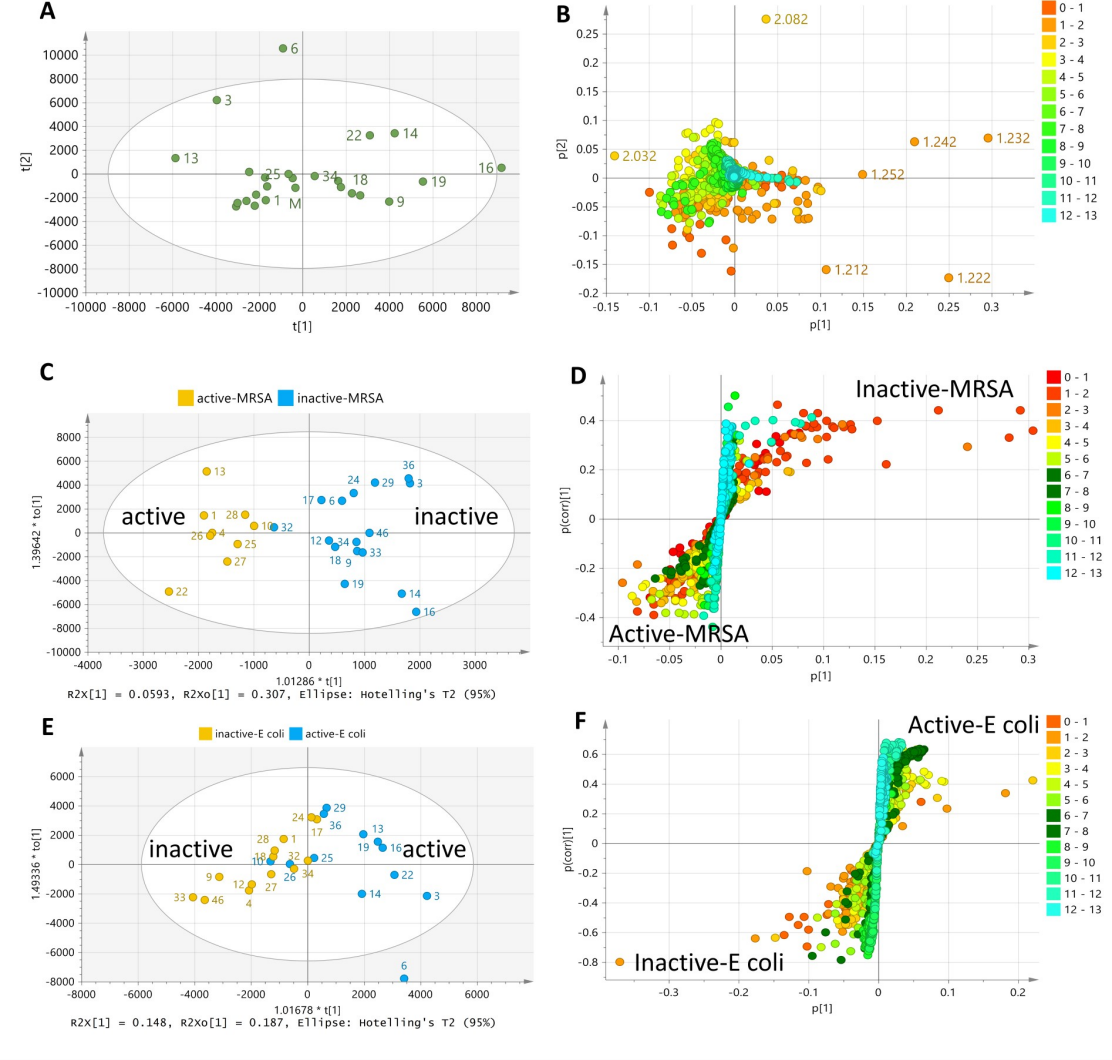
Multivariate analysis of the ^1^H-NMR chemical shift data in ppm of 25 extracts tested against MRSA and *E*. *coli*. (**6A**) PCA-scores and (**6B**) -loadings plot of 25 isolates. OPLS-DA scores plot against MRSA (**6C**) and *E*. *coli* (**6E**). OPLS-DA loadings S-plot of isolates against MRSA (**6D**) and *E*. *coli* (**6F**).

PCA of the proton NMR data of the 25 bioactive isolates achieved R^2^ and Q^2^ values of 0.84 and 0.59, respectively from 5 components. PCA-scores plot (Fig 6A) of the proton NMR data showed a comparable result to the PCA results of the HRMS data exhibiting the chemical uniqueness of isolates MS.REE. 3, 6, and 13, which were withdrawn from the rest of the isolates establishing the main cluster. However, the clustering of the isolates was less dense in the center of the ellipse when compared to the PCA plot of the HRMS data. Isolates on the right quadrant were more distant from the media, particularly MS.REE. 9, 14, 16, 19 and 22. MS.REE. 6 and 16 were indicated as outliers. On the scores plot of the HRMS data (Fig 2B), MS.REE. 16 was not an outlier but instead clustered together with the media. The chemical shifts causing the significant separation of the outliers and the other isolates were indicated in the loadings plot (Fig 6B). Isolates MS.REE. 3, 6, and 13 on the left upper quadrant were dominated by the signals 2–5 ppm, which indicated acetylated and/or glycosidic metabolites. While on the right quadrant represented by isolates MS.REE. 9, 14, 16, 19 and 22 were presided with chemical shifts between 0 and 2 ppm directed towards the presence of saturated fatty acids in the aforementioned isolates. Aromatic signals between 6 and 12 ppm were found on the center of the ellipse where the media was also positioned.

Both the outlying isolates MS.REE. 6 and 16 did not exhibit any bioactivity against MRSA. OPLS-DA plots (Fig 6C and 6D) of the bioactivity of isolates against MRSA, similarly, indicated the chemical uniqueness of isolates MS.REE. 13 and 22 as determined from the multivariate analysis of the HRMS data. MS.REE. 13 and 22 were situated at the end of the active cluster on the left quadrant of the scores plot (Fig 6C), positioned at the upper and lower quadrants, respectively. The internodal variation score within the group was at 30.7% while the variation score between the groups was at 5.93% as the isolates for both groups were distributed on both the upper and lower quadrants of the scores plot. The S-plot shown on Fig 6D exhibited chemical shifts between 3 and 10 ppm for the active end while the inactive end was dominated by aliphatic signals ranging from 0 to 2 ppm.

OPLS-DA plots (Fig 6E and 6F) for the bioactivity of the isolates on *E*. *coli* suggested MS.REE. 3, 6, 14, and 22 as the most distinct in terms of their chemical profile as shown by the scores plot on Fig 6E. However, the S plot on Fig 6F did not indicate a clear separation of the type of chemical shifts between the active and inactive group, although a higher density of the aromatic signals (6–8 ppm) can be observed on the active end. This was also indicated by the overlapping of the active and inactive isolates on the scores plot resulting to a small difference in variation scores within and between groups at 18.7% and 14.8%, respectively.

### Dereplication of the selected isolates

As mentioned above, two outlying isolates MS.REE. 13 and 22 were found highly active against all bacterial strains tested. Isolates MS.REE. 13 and 22 were then revisited and investigated intensively in terms of their dereplication results. Both isolates yielded a high number of *m/z* features for their respective extracts (S1 Table). The total number of features combined from both positive and negative ionization mode for MS.REE. 13 was 8885, which was a little bit more than MS.REE. 22, which had 8831 features. About 48% of the features detected in MS.REE. 13 were known metabolites while 52% afforded no matching hits from the DNP database. This result was very similar to MS.REE. 22, which had 47% of its features as known compounds while the remaining 53% had no hits in the DNP database (S1 Table). This dereplication result indicated a higher occurrence of novel metabolites from both selected isolates as more than 50% of their metabolites afforded no matching hits from the DNP database. Table 2 summarizes the dereplication of the interesting metabolites indicated on the active side of the S-plot for the anti-MRSA and anti-*E*. *coli* activities with highest peak intensities occurring either MS.REE. 13 or MS.REE. 22. Most of the *m/z* features in Table 2 were unidentified from the DNP database, which are promising for novel natural products discovery. Compounds with matching hits from the DNP database listed in Table 2 were putatively identified as earlier reported from microbial natural products.

**Fig 7:**
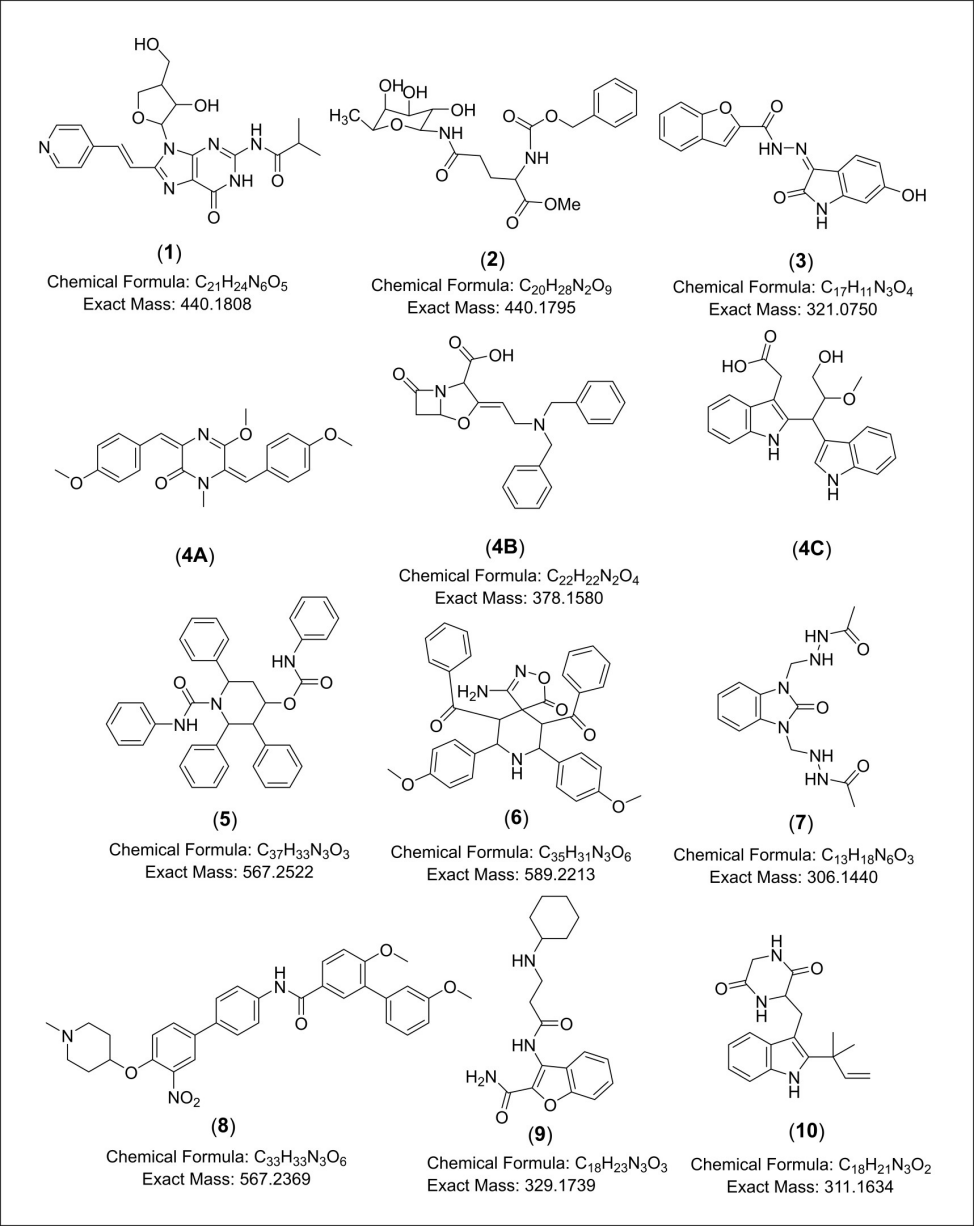
Chemical structures of dereplicated bioactive metabolites for the selected isolates MS.REE. 3, 6, 13 and 22.

Regarding isolate MS.REE. 13, as shown in S2 Table, all the predicted molecular formula of the major ion peaks exhibited a high number of oxygen atoms or the presence of both oxygen and nitrogen moieties, which indicated the occurrence of either glycosidic compounds or peptides. This matched the result of NMR loadings plot where isolate MS.REE. 13 afforded higher density of chemical shifts in the 3 to 5 ppm region indicated by the yellow dots as shown on the left upper quadrant of the loadings plot in Fig 6B. This can be confirmed from the COSY correlation spectrum of MS.REE. 13 that when overlaid with that of the culture medium, it indicated peaks at 3 to 5 ppm exclusively from isolate 13 (S2 Fig). Dereplication study showed that most of the higher intensity ion peaks of MS.REE. 13 (S3A and S3B Fig) yielded hits from the DNP database (S2 Table). However, some of the matching hits of the identified metabolites were of fungal origin and not from actinomycetes while only two hits were putatively identified as bacterial metabolites. Most of the higher intensity ion peaks from isolate MS.REE. 13 were also indicated on the bioactive end of the S-plot. The dereplication of the classified bioactive compounds afforded some interesting hits that included ion peak at *m/z* 379.165 [M+H]^+^, with a predicted molecular formula of C_22_H_22_N_2_O_4._ which was putatively identified as bacterial metabolites **4A**, **B**, or **C**. This ion peak was putatively identified as either nocazine A (**4A**), a metabolite isolated from the marine actinomycete *Nocardiopsis dassonvillei* HR10-5 [66]. This feature was also putatively identified as 2'-deoxy,2'-dibenzylamino clavulanic acid (**4B**), a derivative of clavulanic acid which was first described to be produced by *Streptomyces clavuligerus* (*S*. *clavuligerus*) ATCC 27064 as a β-lactamase inhibitor [67] or (2-[3-hydroxy-2-methoxy-1-(1*H*-indol-3-yl)propyl]-1*H*-indole-3-acetic acid) (**4C**), an actinobacterial metabolite isolated from marine-derived actinomycete *Rubrobacter radiotolerans* [68] (Fig 7). The MS fragmentation of the ion peak at *m/z* 379.165 [M+H]^+^ with a retention time of 10.66 min, which was among the major peaks observed from the base peak chromatogram of MS.REE. 13 **(**S6 Fig) was compatible to that of **4C**. Another ion peak at *m/z* 312.170 [M+H]^+^ with a predicted formula of C_18_H_21_N_3_O_2_ was putatively identified as the fungal metabolite talathermophilin E (**10**), which was isolated from fungus *Talaromyces thermophilus* strain YM3-4 [69] (Fig 7).

Through inspection of the HRMS chromatogram of MS.REE. 22, which was overlaid with the chromatogram of the culture medium, it was observed that many of the major ion peaks did belong to MS.REE. 22 in both positive and negative ionization modes (S4A and S4B Fig). Listed ion peaks shown in S3 Table were mostly higher intensity peaks that did not yield any hits from the DNP database, which is quite promising to go for further scale-up chemical isolation work. It was also interesting to note that the RDBs of the predicted molecular formula of most of the higher intensity peaks observed in MS.REE. 22 were between 5 and 13 ppm indicating the presence of aromatic and/or olefinic compounds. The presence of the aromatic and/or olefinic compounds was also demonstrated by the results shown in Fig 6D. Target bioactive features for isolate MS.REE. 22 on the NMR loadings plot (Fig 6D) exhibited chemical shifts at 5–7 ppm and were also highlighted in the COSY correlation spectrum as shown in S5 Fig. As found in isolate MS.REE. 13, isolate 22 afforded a similar major ion peak at *m/z* 379.165 [M+H]^+^.

Other ion peaks that were detected on the active end of the S-plots but were not matched to any compounds from the DNP included ion peaks at *m/z* [M+H]^+^ 312.170, 322.081, 330.181, 379.165, 441.181, 568.244, and 590.226, while for the negative mode, ion peaks at *m/z* [M-H]^-^ 204.071, 305.138, and 566.245 were found. Except for the ion peak at *m/z* 204.071 [M-H]^-^, all ion peaks generated a predicted molecular formula and the putatively identified bioactive metabolites contained both nitrogen and oxygen with RDBEs between 8 to 23. Using Chemspider, the predicted molecular formulas with the search term “antimicrobials”, synthetically produced compounds were dereplicated, which included 8-substituted-2’-deoxyguanosines (**1**) [70], glycosyl amides (**2**) [71], benzofurans (**3**, **9**) [72, 73], diphenyl-4-hydroxypiperidines (**5**) [74], spiro heterocycles (**6**) [75], benzimidazoles (**7**) [76] and biphenyls (**8**) [77]. N_2020 or compound **7** was dereplicated as an antimicrobial synthetic benzimidazole congener [76] and was a metabolite determined to be putatively specific against *E*. *coli*. Synthesized benzimidazoles have been lately described for their antibacterial activities similarly against *S*. *aureus* and *E*. *coli* [78].

The dereplication work done in this study, was used to assist the isolation of the bioactive metabolites from the selected isolates. From the 12 putatively dereplicated targeted bioactive compounds listed on Table 2 in this study, only four were natural products (**4A**, **4B**, **4C**, and **10**) that were previously described from Actinomycetes, while solely metabolite **4B** (2'-deoxy clavulanic acid) was the only one reported to be isolated from a *Streptomyces* species. All these putatively identified known natural products were dereplicated from both MS.REE. 13 and 22, with higher concentrations found in MS.REE. 13. S2 Table and S3 Table listed the 6 major peaks for each isolates including those two ion peaks identified to be the bioactive metabolites **4A**, **4B** or **4C**, and **10**. However, the other two ion peaks dereplicated for isolate 13 were described as fungal metabolites while two other peaks did not give any matching hit. For isolate 22, the rest of the major ion peaks did not find any matching hit from the DNP database. This dereplication step indicated the uniqueness of the chemical profiles of MS.REE. 13 and 22 with novel natural products to be subjected for further chromatographic isolation work. However, it was also important and necessary to explore the type of bioactive compounds that will match the predicted molecular formula from a database like Chemspider. The set of information provided by the dereplication study, specifically those hits found from Chemspider albeit chemically synthesized compounds was still a useful aid in determining the mode of isolation work and structural elucidation tools needed to identify the bioactive metabolites. The deduced information from Chemspider on the type of the expected chemistry for these new bioactive natural products were compatible with the NMR spectral data as already mentioned above and as shown in S2 Fig as well as indicated by the NMR loadings plot (Fig 6D and 6F) for focusing into the functional groups responsible for the bioactivity of the predicted metabolites. The dereplication work presented here will not be conclusive in terms of elucidating the structure of the compounds but the objective of this study is to put together a systematic methodology that would be statistically robust in prioritizing isolates from an underexplored collection of microorganisms for further tedious chemical work in finally identifying the antibacterial compound. The results of this dereplication study coupled to a metabolomics approach by utilizing multivariate analysis has provided the practicality of targeting and pinpointing the bioactive metabolites as also shown by our earlier studies [17, 22, 29–37]. Such a coupled approach has proven to be time and cost-efficient when the target bioactive metabolites were directly pinpointed and partially elucidated from the first fractionation stage.

**Fig 8:**
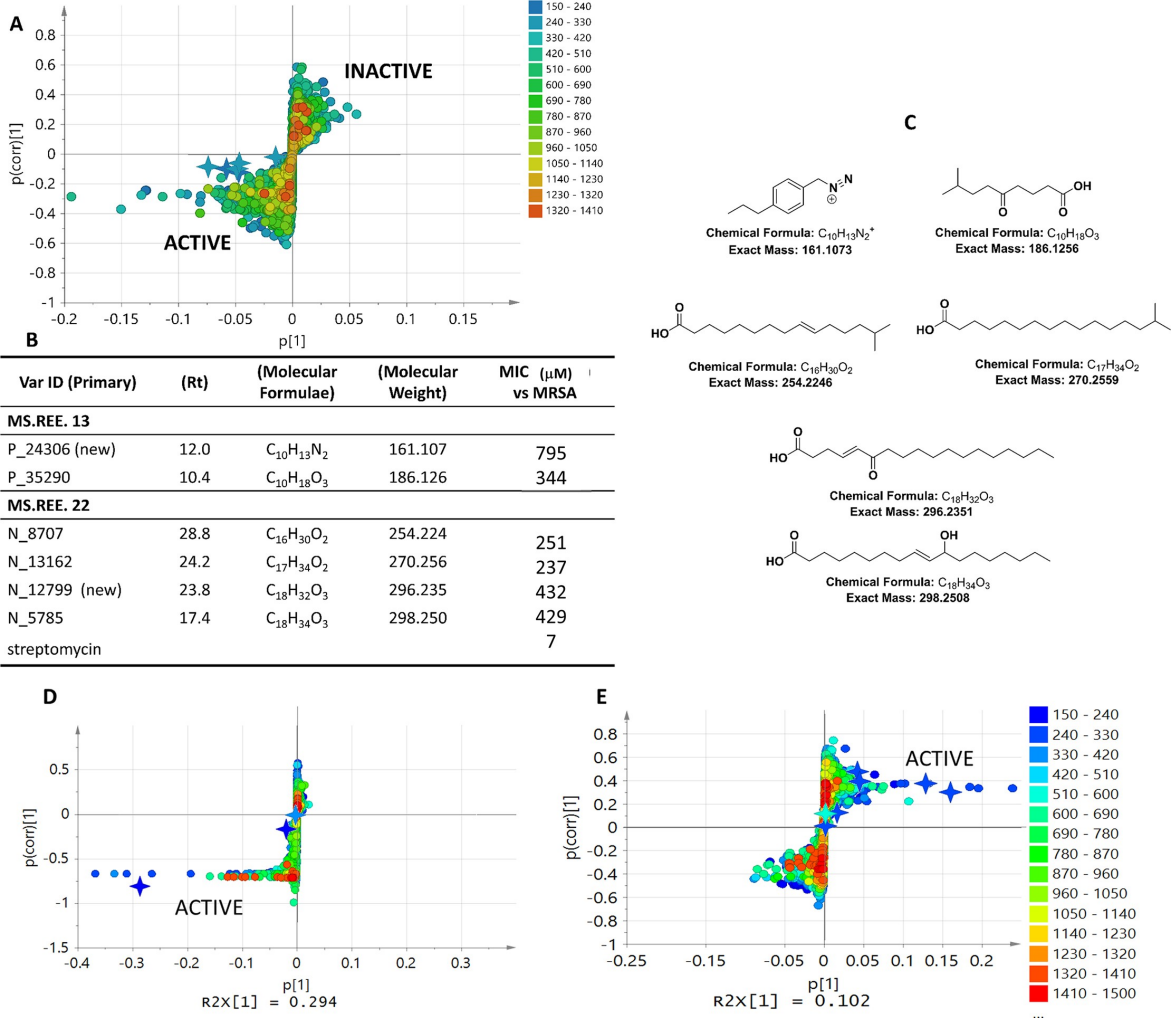
Mapping the targeted and isolated bioactive metabolites on OPLS-DA plots. **(8A)** OPLS-DA loadings plot of crude extracts. The isolated compounds are represented by a 4-point star. **(8B)** Dereplication table and bioassay results against MRSA for the isolated compounds with streptomycin as positive standard. **(8C)** Structures of the isolated bioactive compounds. **(8D)** OPLS-DA loadings plot of MS. REE. 13 fractions. **(8E)** OPLS-DA loadings plot of MS. REE. 22 fractions. Both isolated and the targeted bioactive metabolites were marked with a 4-point star.

The utilization of MVA was shown to identify the bioactive metabolite regardless of its concentration in the fraction of the crude extract by using the OPLS-DA loadings S-plot. The metabolites were distributed according to the same quadrants to where the active and inactive fractions were positioned on the OPLS-DA scores plots. This approach rectified the common oversight in a bioassay-guided isolation procedure where the major components of a bioactive crude extract or fraction were more perceptibly targeted to afford a pure but inactive metabolite. In this study, it was demonstrated that the major ion peaks detected on the base peak chromatogram (S3A and S3B Fig, and S4A and S4B Fig) were not designated as the bioactive metabolite in the OPLS_DA loadings S-plot. The ion peaks were not detectable in the base peak chromatogram either due to their low yield or poor ionization capability. It is, indeed, however a challenge when a metabolomics-guided approach is utilized to target a bioactive metabolite that is present in a crude extract or fraction at nanogram levels, which could be unfeasible to isolate. In addition, the relative concentration of the ion peaks on the total ion chromatogram is highly dependent on the ability of the respective compounds to ionize at certain modes and methods of ionization, for this reason, the NMR spectral data does support the analysis for the “true” relative abundance of the various metabolites. The isolated compounds afforded medium to weaker antibiotic activity with MICs from 250 to 800 μM. Albeit, the occurrence of these isolated compounds was apparent only on the active quadrant of the OPLS-DA loadings plot of the crude extracts (Fig 8A–8C) and not found in the inactive side of the plot. Two new compounds, P_24306 (C_10_H_13_N_2_) and N_12799 (C_18_H_32_O_3_) with MICs of 795 and 432 μM, were afforded from the scale-up of MS.REE. 13 and 22, respectively (Fig 8B and 8C). Fractionation of the scaled-up isolates MS.REE. 13 and 22 did reveal the targeted bioactive metabolites listed on Table 2 along with the isolated compounds on the active quadrant of the OPLS-DA plot grouped according to their activity against MRSA. It was also demonstrated that the isolated compounds were not easily ionized in the electro-spray ionization mass spectral platform resulting to the low intensity of their mass ion peaks although the corresponding proton signals can be detected by NMR (Fig 6D) with resonances between 0–3 ppm for the acetylated aliphatic alkyl chain and 4–6 ppm for the hydroxylated and olefinic moieties. The mass ion peak intensities of the isolated compounds did improve preceding fractionation. A bioassay- and metabolomic-guided approach was also used to chromatographically isolate the bioactive metabolites shown on Fig 8D and 8E. Subsequent to fractionation, the detected bioactive metabolites from the crude extracts were still positioned on the active quadrant. However, it was also observed that a new set of bioactive metabolites were also detected. As the concentration of the less ionized bioactive metabolites increased, it was feasible to go over its threshold level for quantification.

### Molecular biological characterization of the selected actinomycete isolates

**Fig 9:**
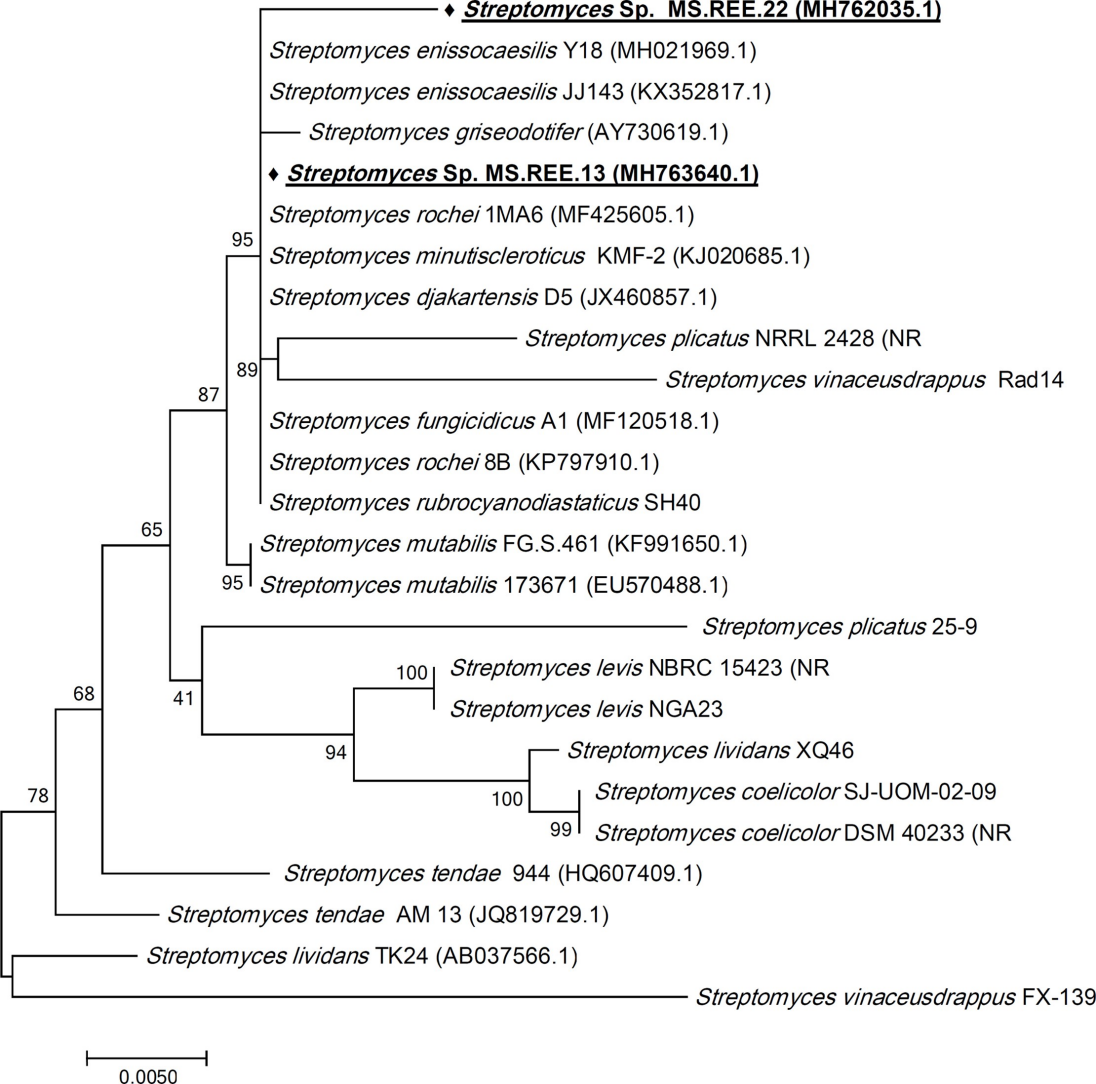
Phylogenetic tree of MS.REE. 13 and MS.REE. 22 isolates based on partial 16S rRNA gene sequences. The phylogenetic tree was inferred using the Neighbor-Joining method [81]. The distances were computed using the Kimura 2-parameter method [82] and are in the units of the number of base substitutions per site. Numbers at nodes indicate percentages of 1000 bootstrap re-samplings. The analysis involved 25 nucleotide sequence. Codon positions included were 1st+2nd+3rd+Noncoding. All positions containing gaps and missing data were eliminated. Evolutionary analyses were conducted in MEGA7 [83].

The amplified 16S rRNA gene sequences of the selected isolates MS.REE. 13 and 22 showed very high similarity (100% and 99% respectively) with many *Streptomyces* sp. sequences in NCBI GenBank. The partial 16S rRNA gene sequences of MS.REE. 13 and 22 aligned with closely related sequences from the NCBI GenBank and by using MEGA7 software, the phylogenetic tree of the isolates was created using the Neighbor-Joining method. As shown in Fig 9, many Streptomyces hits were similar to our selected isolates which confirmed that they belong to the genus Streptomyces. However, it was difficult to assign the taxonomical classification to the species level. Therefore, both isolates were classified as *Streptomyces* sp., which is consistent with an earlier report [79] mentioning that 16s rRNA gene sequencing is an insufficient tool to assign the phylogeny of the closely related species because of the conserved sequences. The results of the present study partially matches earlier studies [80] as they managed to define the phylogenetic identification of many *Streptomyces* sp. using the 16S rRNA sequencing of many strains, however it was not a useful tool to identify some other strains to species level in the same study.

**Fig 10:**
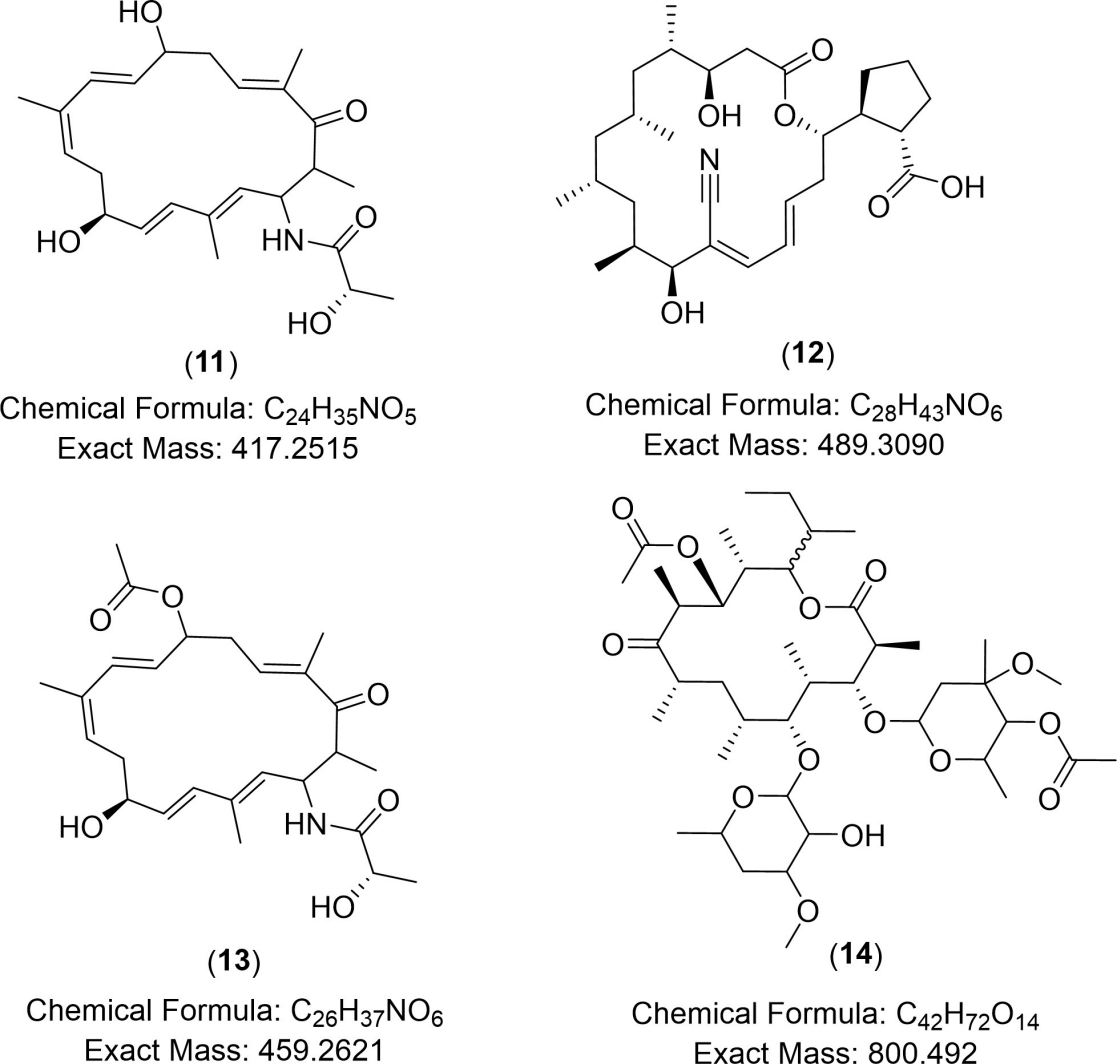
Chemical structures of dereplicated metabolites earlier reported from related species *S*. *rochei*.

The closely related actinomycete *S*. *rochei* have the biggest number of 37 reported natural products with 15 compounds described to exhibit antimicrobial activity (antibacterial or antifungal). Interesting metabolites were detected in the selected isolates MS.REE. 13 and 22 that were earlier isolated from *S*. *rochei* which was the only related species producing these dereplicated metabolites, while the other species neighbor to our selected isolates did not afford any matching compounds in the dereplication study. Five putatively identified metabolites were earlier described from *S*. *rochei*, from which four compounds were detected from MS.REE. 22 and three metabolites were found in MS.REE. 13 with two shared compounds produced by both isolates (Table 3, Fig 10). One of the interesting hits was the ion peak at *m/z* 418.259 [M+H]^+^ that was yielded from MS.REE. 13, with a molecular formula of C_24_H_35_NO_5_, which was putatively identified as lankacyclinol (**11**). Lankacyclinol (**11**) or also known as antibiotic T 2636G is a polyene-type antibiotic that exhibited potent antimicrobial activity [84, 85]. Furthermore, another ion peak at *m/z* 490.316 [M+H]^+^ with predicted molecular formula of C_28_H_43_NO_6_ that was detected in both isolates MS.REE. 13 and 22 was putatively identified as borrelidin (**12**), an antiviral agent also isolated from *S*. *rochei* [86, 87]. The MS fragmentation of the ion peak at *m/z* 490.316 [M+H]^+^ is shown on S7 Fig that is compatible for borrelidin (**12**). On the other hand, another derivative with a molecular formula of C_26_H_37_NO_6_ was found at *m/z* 460.270 [M+H]^+^, which was only produced by MS.REE. 22 was putatively identified as lankacyclinol A (**13**) also named as antibiotic T 2636E [85]. Another ion peak detected in MS.REE. 22 was found at *m/z* 801.501 [M+H]^+^ with a molecular formula of C_42_H_72_O_14_ and was identified to be 8,15-dideoxylankamycin (**14**), a compound isolated from a mutant strain of *S*. *rochei* [88]. This metabolite was a derivative of the macrolide antibiotic lankamycin [88, 89]. The MS fragmentation of the ion peak at *m/z* 801.501 [M+H]^+^ is presented on S8 Fig that is well-matched for 8,15-dideoxylankamycin (**14**). Due to the observed lower intensity ion peaks at *m/z* [M+H]^+^ 418.259 and 460.270, it was not plausible to deduce any MS fragmentation data. These dereplication results were essential to further confirm chemotaxonomical relationship of the bioactive isolates found in this current collection to the strain *S*. *rochei*. Although chemical biomarkers specific for *S*. *rochei* were found and dereplicated, these compounds were not necessarily responsible for the bioactivity of the crude extracts as indicated in this study. For this two-year research project, due to time and funding constraints, it was only possible to subject two isolates for taxonomical identification and hence, it was vital to set-up this metabolomics-guided dereplication approach in prioritizing the isolates for further work. Taxonomical work would entail full data analysis for the biochemical identification, morphological characterization, 16S rRNA and DNA-DNA hyperdization.

**Table 3:** Dereplicated metabolites, which were earlier reported from related species *S*. *rochei* as detected in the phylogenetic analysis of the bioactive isolates MS.REE. 13 and 22. Structures of compounds 11 to 14 are shown in Fig 10.

MZmine ID	RT	*m/z*	MW (accuracy in ppm)	Predicted Molecular formula	Putative compound identified	Isolate yielding the metabolite
**N_ 2571**	16.20	656.270 [M-H]^-^	657.278 (-0.64)	C_34_H_43_NO_12_	antibiotic T 2636K	13 22
**P_ 22661**	18.58	418.259 [M+H]^+^	417.251 (-1.15)	C_24_H_35_NO_5_	lankacyclinol (**11**)	13
**P_ 7908**	19.03	490.316 [M+H]^+^	489.309 (0.007)	C_28_H_43_NO_6_	borrelidin (**12**)	13 22
**P_32128**	21.29	460.27 [M+H]^+^	459.262 (1.35)	C_26_H_37_NO_6_	lankacyclinol A (**13**)	22
**P_ 33188**	23.42	801.501 [M+H]^+^	800.492 (2.09)	C_42_H_72_O_14_	8,15-dideoxylankamycin (**14**)	22

## Conclusions

A total of 58 isolates were isolated from different soil samples collected from Ihnasia City, Egypt from which 25 isolates were active against at least one of the tested indicator microbial strains. Chemical profiles of the bioactive crude extracts were investigated using both NMR and LC-HRMS followed by multivariate analysis which was performed for 25 bioactive extracts. PCA was used to differentiate the isolates depending on their chemical diversity and novelty, while OPLS-DA was used to compare the chemical profiles of the NMR and mass spectral data of the isolates by classifying the extracts and fractions according to their biological activity. The tested isolates exhibited a chemical diversity of 84% indicating four outlying extracts MS.REE. 3, 6, 12, and 22 by PCA. MS.REE.13 and 22 were chosen for scale-up work. On the other hand, outliers MS.REE. 3 and 6, which were only found active against *E*. *coli* and inactive against MRSA that was the main target activity for initial scale-up work, were then excluded for further analysis. Dereplication with a metabolomics approach was a fascinating tool used for isolate selection and prioritization as it helped in predicting the probability of each isolate to produce novel compounds, the functional groups responsible for the bioactivity, and to know the source of each metabolite which was preliminarily identified putatively. By correlating the dereplication results with the biological activity of the isolates, it was demonstrated that selection of isolates which were both biologically active and had interesting chemical profiles could support the efforts of the drug discovery campaign and increases the chance for the discovery of novel natural products. The metabolomics approach was a valuable tool in isolate prioritization for drug discovery programs due to the low chance of redundancy of isolating the same set of compounds with similar reported bioactivities. A metabolomics-guided approach provided the lead to targeting bioactive metabolites that are present in a crude extract or fraction even at nanogram levels, which may not be perceivable in a chromatogram, but it is, however, a challenge that such low yields would be feasible to isolate. The isolated compounds exhibited medium to weak antibiotic activity with MICs from 250 to 800 μM while their occurrence was established to be only on the active side of OPLS-DA loadings plot (Fig 8). Two new compounds, P_24306 (C_10_H_13_N_2_) and N_12799 (C_18_H_32_O_3_) with MICs of 795 and 432 μM, were afforded from the scale-up of MS.REE. 13 and 22, respectively. Most active were (*E*)-14-methylpentadec-9-enoic acid and 15-methylhexadecanoic acid with MIC values of approximately 250 μM due to their enhanced capability to permeate the lipophilic cell membrane [90]. The pinpointed bioactive natural products (P<0.01) through a metabolomics approach, particularly those established by mass spectrometry, will have to be revisited to enhance their production and yield, which would need optimization of the culture media by changing the carbon or nitrogen sources or the addition of elicitors.

However, as realized in this study, it is important to use a combination of analytical tools to target all types of diverse chemistry. Such analytical tools must also include a combination of different ionization platforms for mass spectrometry albeit NMR spectroscopy does fill up the gap in getting the entire representation of the different ratios of the various compounds present in a mixture, the presence of metabolites at micro- or nanogram levels is not feasible to detect. From this study, it was further concluded that Egyptian soil actinomycetes samples were found to be still a rich source of biologically active natural products worth for further research.

## Supporting information

S1 Fig Antimicrobial activity screening of crude extracts using cup diffusion method against *B*. *subtilis* as illustrated for MS.REE. 1, 3, 4, and 6. The zones of inhibition were measured and recorded as active when it was greater than 12 mm. Twenty-five bioactive isolates were found out of the total of 58 isolated actinomycetes.
(DOCX)

S2 Fig 2D-NMR COSY spectrum of MS.REE. 13 overlaid with culture medium. Signals in orange are from the MS.REE. 13 and signals in grey are from the culture medium. Highlighted correlations indicate the significant chemical shifts which made MS.REE. 13 an outlier.
(DOCX)

S3 Fig Base peak chromatograms of both positive and negative modes for the bacterial extract of MS.REE. 13, annotated to indicate major metabolites produced by the bacteria and are not from the medium shown in S2 Table. **(A)** Positive ion mode and **(B)** Negative ion mode.
(DOCX)

S4 Fig Base peak chromatograms of both positive and negative modes for the bacterial extract of MS.REE. 22, annotated to indicate major metabolites produced by the bacteria and are not from the medium shown in S3 Table. **(A)** Positive ion mode and **(B)** Negative ion mode.
(DOCX)

S5 Fig 2D-NMR COSY spectrum of MS.REE. 22 overlaid with culture medium. Signals in orange are from the MS.REE. 22 and signals in grey are from the culture medium. Highlighted correlations indicate the significant chemical shifts which made MS.REE. 22 an outlier.
(DOCX)

S6 Fig HRMS fragmentation of the ion peak at *m/z* 379.165 [M+H]^+^ for 2-[3-hydroxy-2-methoxy-1-(1H-indol-3-yl) propyl]-1H-indole-3-acetic acid (4C). (DOCX)

S7 Fig HRMS fragmentation of the ion peak at *m/z* 490.316 [M+H]^+^ for borrelidin (12). (DOCX)

S8 Fig HRMS fragmentation of the ion peak at *m/z* 801.501 [M+H]^+^ for 8,15-dideoxylankamycin (14). (DOCX)

S1 Table Summary of the total number of features (*m/z*) detected in the bioactive outlying bacterial extracts (MS.REE. 13 and 22) including number of features in negative and positive modes after solvent peaks removal (a), number of features after removing the effect of the culture medium (b) and number of both unknown and putatively identified features from DNP database (c).
(DOCX)

S2 Table Dereplication of selected major ion peaks in MS.REE. 13. (DOCX)

S3 Table Dereplicated selected major ion peaks in MS.REE. 22. (DOCX)

S4 Table Different seasons during which the bioactive isolates were collected. (DOCX)
