# Correction: Bioassay- and metabolomics-guided screening of bioactive soil actinomycetes from the ancient city of Ihnasia, Egypt

**DOI:** 10.1371/journal.pone.0228901

**Published:** 2020-02-03

**Authors:** Mohamed Sebak, Amal E Saafan, Sameh AbdelGhani, Walid Bakeer, Ahmed O El-Gendy, Laia Castaño Espriu, Katherine Duncan, RuAngelie Edrada-Ebel

The second affiliation for the first author is incorrect. Mohamed Sebak is not affiliated with #2 but with #3: Microbiology and Immunology Department, Faculty of Pharmacy, Beni-Suef University, Beni-Suef, Egypt.
