# Supplementary material for: Bioassay- and metabolomics-guided screening of bioactive soil actinomycetes from the ancient city of Ihnasia, Egypt
Source: PLoS One. 2019 Dec 30;14(12):e0226959. doi: 10.1371/journal.pone.0226959 (PMC6936774; doi:10.1371/journal.pone.0226959)
Supplement: S1 Fig — The zones of inhibition were measured and recorded as active when it was greater than 12 mm. Twenty-five bioactive isolates were found out of the total of 58 isolated actinomycetes. (DOCX) [file pone.0226959.s001.docx]

Supporting Information

**Bioassay- and Metabolomics-guided Screening of Bioactive Soil Actinomycetes from the Ancient City of Ihnasia, Egypt**

**Mohamed Sebak ^1,2,*^, Amal E. Saafan^2^,** **Sameh AbdelGhani^2^, Walid Bakeer^2^, Ahmed O. El-Gendy^2^, Laia Castaño Espriu^1^, Katherine Duncan^1^,** **RuAngelie Edrada-Ebel^1*^**

^1^ Strathclyde Institute of Pharmacy and Biomedical Sciences, Faculty of Science, University of Strathclyde, Glasgow, UK.

^2^ Microbiology and Immunology Department, Faculty of Pharmacy, Beni-Suef University, Beni-Suef, Egypt.

***Correspondence:**

Mohamed Sebak

E-mail: [Mohamed.sebak@pharm.bsu.edu.eg](mailto:Mohamed.sebak@pharm.bsu.edu.eg)

RuAngelie Edrada-Ebel

E-mail: [Ruangelie.edrada-ebel@strath.ac.uk](mailto:Ruangelie.edrada-ebel@strath.ac.uk)


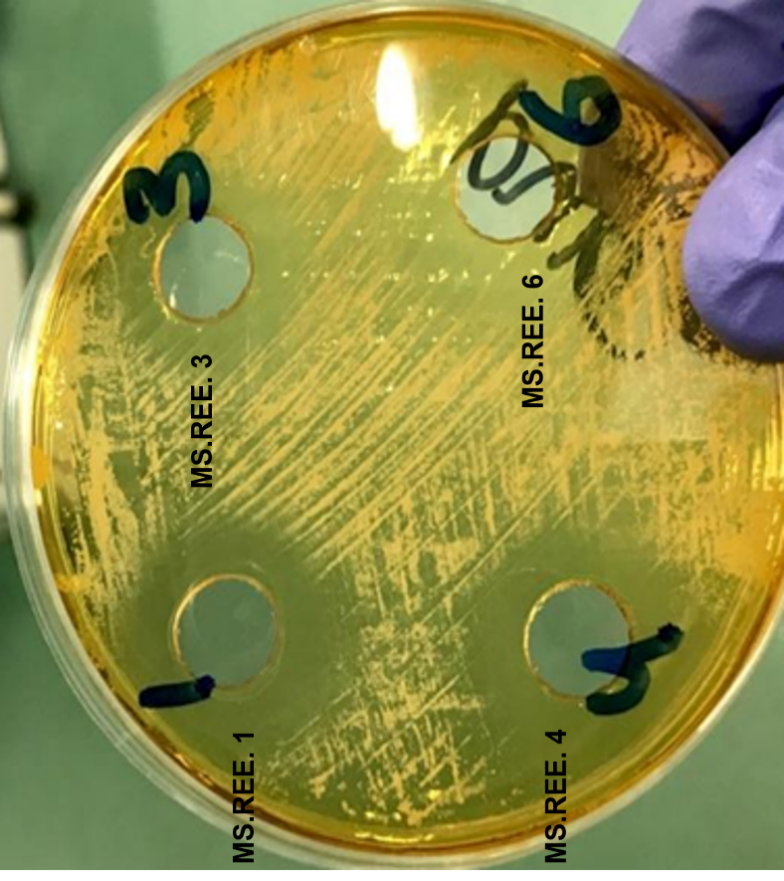


**S1 Fig. Antimicrobial activity screening of crude ethyl acetate extracts using cup diffusion method against *B. subtilis* as illustrated for MS.REE. 1, 3, 4, and 6.** **The zones of inhibition were measured and recorded as active when it was greater than 12 mm. The cup has a diameter of 10mm. Twenty-five bioactive isolates were found out of the total of 58 isolated actinomycetes.**

Inhibition zones in mm of bioactive outlying extracts classified by multivariate analysis to indicate highest chemical diversity. The antibiotic activity was compared to streptomycin as reference.

| Isolate Extract | vs *B. subtilis* | vs MRSA | vs *E. coli* | vs *Candida albicans* |
| --- | --- | --- | --- | --- |
| MS.REE. 3 | 18 | Inactive | 18 | Inactive |
| MS.REE. 6 | 30 | Inactive | 22 | Inactive |
| MS.REE. 13 | 30 | 19 | 18 | Inactive |
| MS.REE. 22 | 27 | 13 | 18 | Inactive |
| streptomycin | 37 | 25 | 25 | Inactive |
