# Supplementary material for: Bioassay- and metabolomics-guided screening of bioactive soil actinomycetes from the ancient city of Ihnasia, Egypt
Source: PLoS One. 2019 Dec 30;14(12):e0226959. doi: 10.1371/journal.pone.0226959 (PMC6936774; doi:10.1371/journal.pone.0226959)
Supplement: S2 Fig — Signals in orange are from the MS.REE. 13 and signals in grey are from the culture medium. Highlighted correlations indicate the significant chemical shifts which made MS.REE. 13 an outlier. (DOCX) [file pone.0226959.s002.docx]

Supporting Information

**Bioassay- and Metabolomics-guided Screening of Bioactive Soil Actinomycetes from the Ancient City of Ihnasia, Egypt**

**Mohamed Sebak ^1,2,*^, Amal E. Saafan^2^,** **Sameh AbdelGhani^2^, Walid Bakeer^2^, Ahmed O. El-Gendy^2^, Laia Castaño Espriu^1^, Katherine Duncan^1^,** **RuAngelie Edrada-Ebel^1*^**

^1^ Strathclyde Institute of Pharmacy and Biomedical Sciences, Faculty of Science, University of Strathclyde, Glasgow, UK.

^2^ Microbiology and Immunology Department, Faculty of Pharmacy, Beni-Suef University, Beni-Suef, Egypt.

***Correspondence:**

Mohamed Sebak

E-mail: [Mohamed.sebak@pharm.bsu.edu.eg](mailto:Mohamed.sebak@pharm.bsu.edu.eg)

RuAngelie Edrada-Ebel

E-mail: [Ruangelie.edrada-ebel@strath.ac.uk](mailto:Ruangelie.edrada-ebel@strath.ac.uk)


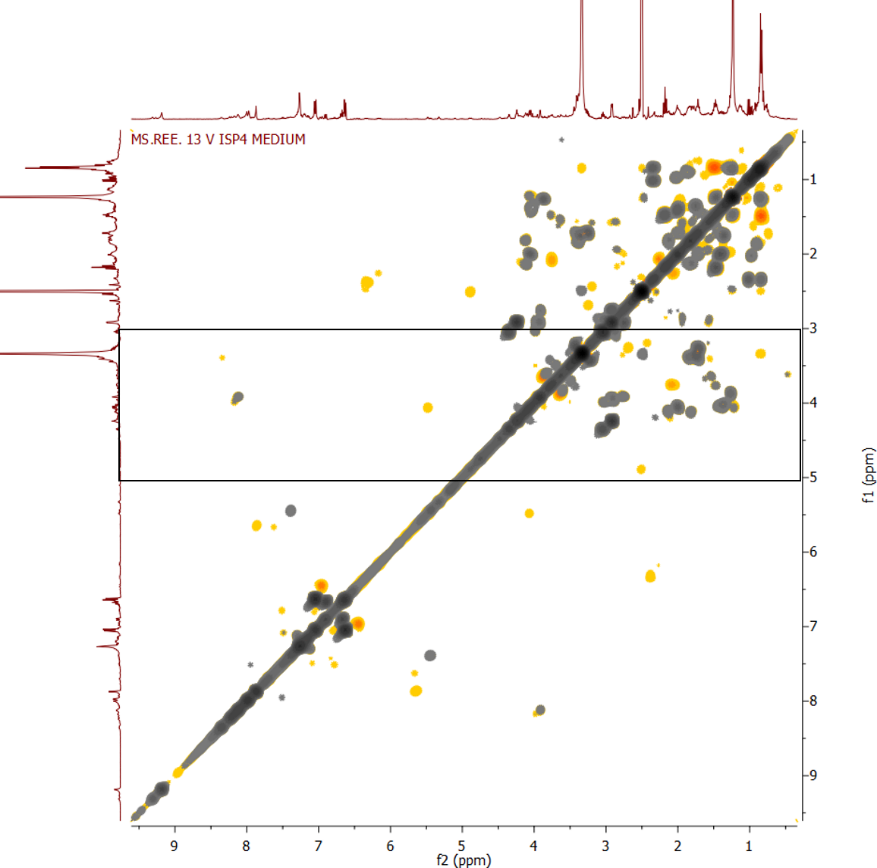


**S2 Fig. 2D-NMR COSY spectrum of MS.REE. 13 overlaid with culture medium.** Signals in orange are from the MS.REE. 13 and signals in grey are from the culture medium. Highlighted correlations indicate the significant chemical shifts which made MS.REE. 13 an outlier.
