# Supplementary material for: Bioassay- and metabolomics-guided screening of bioactive soil actinomycetes from the ancient city of Ihnasia, Egypt
Source: PLoS One. 2019 Dec 30;14(12):e0226959. doi: 10.1371/journal.pone.0226959 (PMC6936774; doi:10.1371/journal.pone.0226959)
Supplement: S3 Fig — (A) Positive ion mode and (B) Negative ion mode. (DOCX) [file pone.0226959.s003.docx]

Supporting Information

**Bioassay- and Metabolomics-guided Screening of Bioactive Soil Actinomycetes from the Ancient City of Ihnasia, Egypt**

**Mohamed Sebak ^1,2,*^, Amal E. Saafan^2^,** **Sameh AbdelGhani^2^, Walid Bakeer^2^, Ahmed O. El-Gendy^2^, Laia Castaño Espriu^1^, Katherine Duncan^1^,** **RuAngelie Edrada-Ebel^1*^**

^1^ Strathclyde Institute of Pharmacy and Biomedical Sciences, Faculty of Science, University of Strathclyde, Glasgow, UK.

^2^ Microbiology and Immunology Department, Faculty of Pharmacy, Beni-Suef University, Beni-Suef, Egypt.

***Correspondence:**

Mohamed Sebak

E-mail: [Mohamed.sebak@pharm.bsu.edu.eg](mailto:Mohamed.sebak@pharm.bsu.edu.eg)

RuAngelie Edrada-Ebel

E-mail: [Ruangelie.edrada-ebel@strath.ac.uk](mailto:Ruangelie.edrada-ebel@strath.ac.uk)


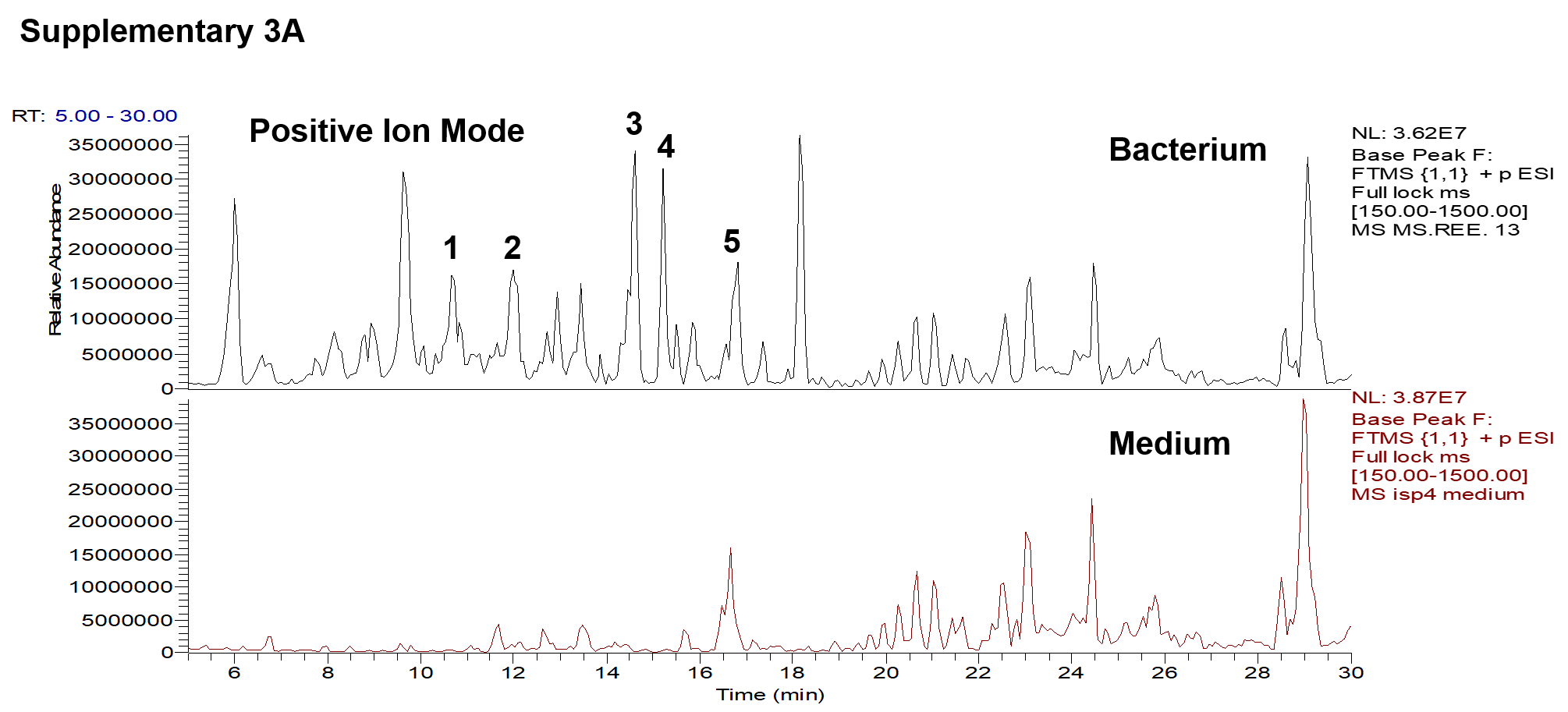

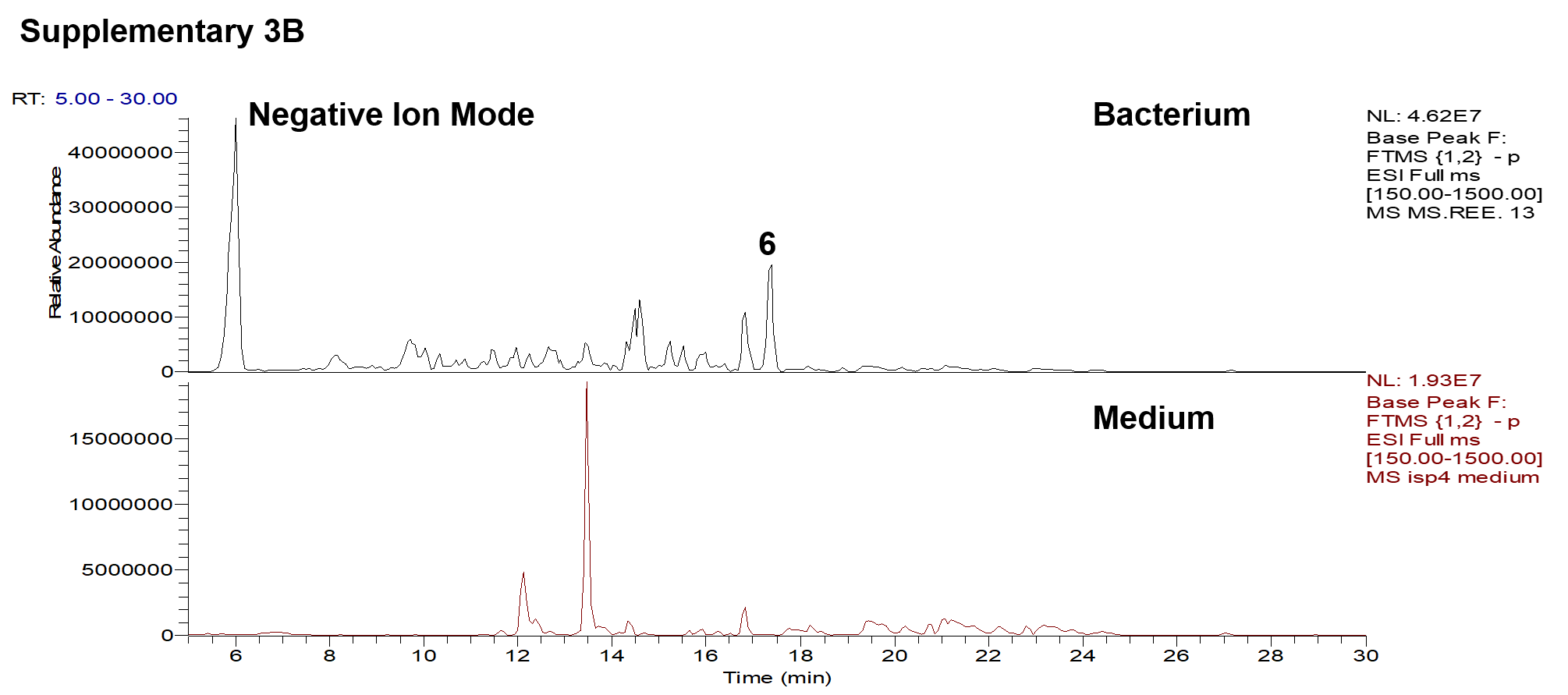


**S3 Fig.** **Base peak chromatograms of both positive and negative modes for the bacterial extract of MS.REE. 13, annotated to indicate major metabolites produced by the bacteria and are not from the medium shown in S2 Table. (A)** Positive ion mode and **(B)** Negative ion mode.
