# Supplementary material for: Bioassay- and metabolomics-guided screening of bioactive soil actinomycetes from the ancient city of Ihnasia, Egypt
Source: PLoS One. 2019 Dec 30;14(12):e0226959. doi: 10.1371/journal.pone.0226959 (PMC6936774; doi:10.1371/journal.pone.0226959)
Supplement: S7 Fig — (DOCX) [file pone.0226959.s007.docx]

Supporting Information

**Bioassay- and Metabolomics-guided Screening of Bioactive Soil Actinomycetes from the Ancient City of Ihnasia, Egypt**

**Mohamed Sebak ^1,2,*^, Amal E. Saafan^2^,** **Sameh AbdelGhani^2^, Walid Bakeer^2^, Ahmed O. El-Gendy^2^, Laia Castaño Espriu^1^, Katherine Duncan^1^,** **RuAngelie Edrada-Ebel^1*^**

^1^ Strathclyde Institute of Pharmacy and Biomedical Sciences, Faculty of Science, University of Strathclyde, Glasgow, UK.

^2^ Microbiology and Immunology Department, Faculty of Pharmacy, Beni-Suef University, Beni-Suef, Egypt.

***Correspondence:**

Mohamed Sebak

E-mail: [Mohamed.sebak@pharm.bsu.edu.eg](mailto:Mohamed.sebak@pharm.bsu.edu.eg)

RuAngelie Edrada-Ebel

E-mail: [Ruangelie.edrada-ebel@strath.ac.uk](mailto:Ruangelie.edrada-ebel@strath.ac.uk)


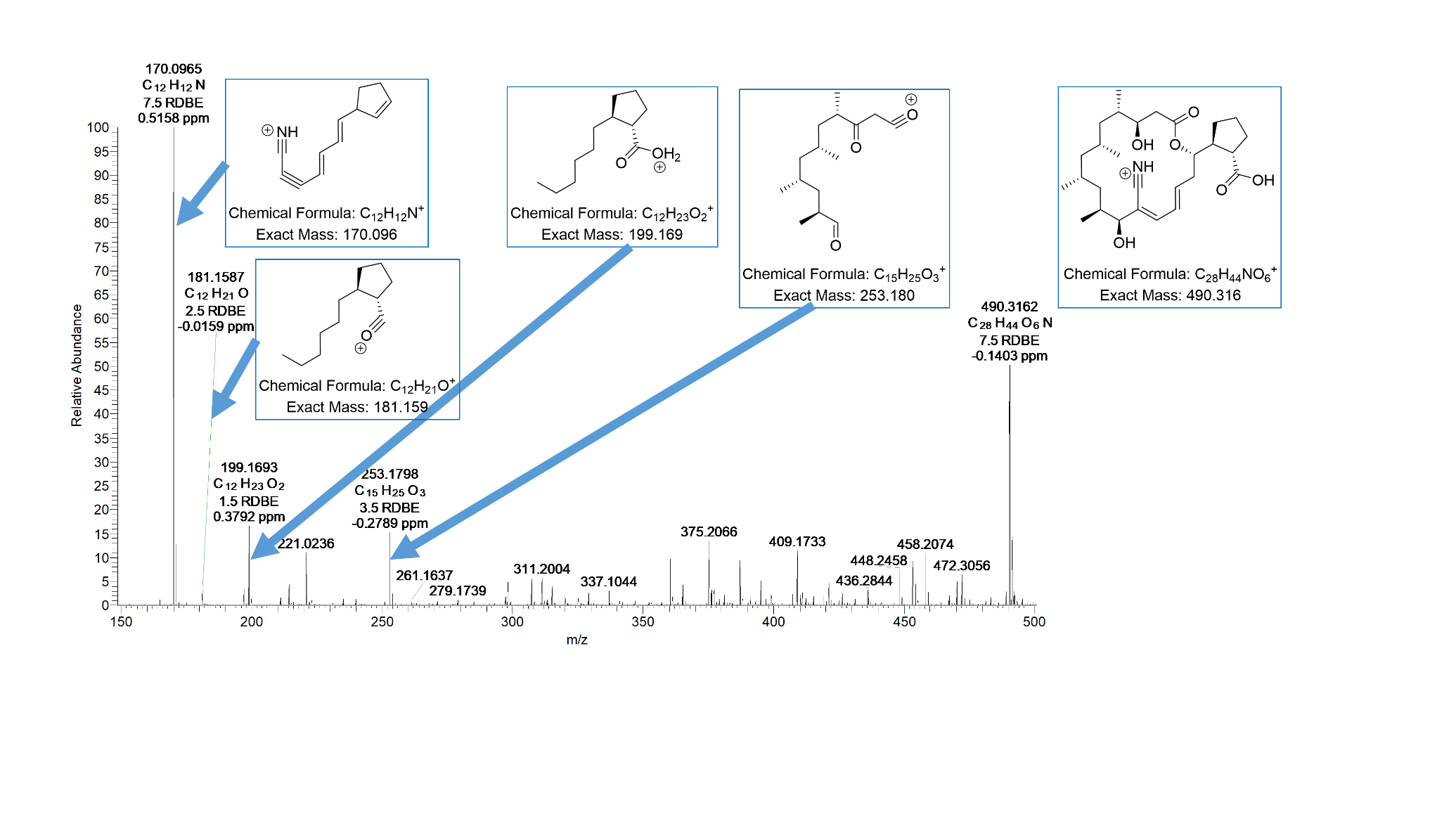


**S7 Fig. HRMS fragmentation of the ion peak at *m/z*490.316 [M+H]^+^ for borrelidin (12).**
