# Supplementary material for: Bioassay- and metabolomics-guided screening of bioactive soil actinomycetes from the ancient city of Ihnasia, Egypt
Source: PLoS One. 2019 Dec 30;14(12):e0226959. doi: 10.1371/journal.pone.0226959 (PMC6936774; doi:10.1371/journal.pone.0226959)
Supplement: S1 Table — Summary of the total number of features (m/z) detected in the bioactive outlying bacterial extracts (MS.REE. 13 and 22) including number of features in negative and positive modes after solvent peaks removal (a), number of features after removing the effect of the culture medium (b) and number of both unknown and putatively identified features from DNP database (c). (DOCX) [file pone.0226959.s009.docx]

Supporting Information

**Bioassay- and Metabolomics-guided Screening of Bioactive Soil Actinomycetes from the Ancient City of Ihnasia, Egypt**

**Mohamed Sebak ^1,2,*^, Amal E. Saafan^2^,** **Sameh AbdelGhani^2^, Walid Bakeer^2^, Ahmed O. El-Gendy^2^, Laia Castaño Espriu^1^, Katherine Duncan^1^,** **RuAngelie Edrada-Ebel^1*^**

^1^ Strathclyde Institute of Pharmacy and Biomedical Sciences, Faculty of Science, University of Strathclyde, Glasgow, UK.

^2^ Microbiology and Immunology Department, Faculty of Pharmacy, Beni-Suef University, Beni-Suef, Egypt.

***Correspondence:**

Mohamed Sebak

E-mail: [Mohamed.sebak@pharm.bsu.edu.eg](mailto:Mohamed.sebak@pharm.bsu.edu.eg)

RuAngelie Edrada-Ebel

E-mail: [Ruangelie.edrada-ebel@strath.ac.uk](mailto:Ruangelie.edrada-ebel@strath.ac.uk)

**S1 Table. Summary of the total number of features (*m/z*) detected in the bioactive outlying bacterial extracts (MS.REE. 13 and 22) including number of features in negative and positive modes after solvent peaks removal (a), number of features after removing the effect of the culture medium (b) and number of both unknown and putatively identified features from DNP database (c).**

| Bacterial Strain | (a)Total features (*m/z*) | | (b) Number of features (*m/z*) after removal ISP4 medium features | | (c) Number of features (*m/z*) identified by dereplication with DNP database | |
| --- | --- | --- | --- | --- | --- | --- |
|  | **Positive Ion Mode** | **Negative Ion Mode** | **Positive**  **Ion Mode** | **Negative**  **Ion Mode** | **Putatively Identified Features (Positive plus Negative mode)** | **Unknown Features (Positive plus Negative mode)** |
| MS.REE. 13 | 17715 | 3944 | 5571  (31.5% remaining) | 3314  (84% remaining) | 4283  (48.2%) | 4602  (51.8%) |
| MS.REE. 22 | 17107 | 3435 | 5980  (35% remaining) | 2851  (83% remaining) | 4147  (47%) | 4684  (53%) |
