# Supplementary material for: Bioassay- and metabolomics-guided screening of bioactive soil actinomycetes from the ancient city of Ihnasia, Egypt
Source: PLoS One. 2019 Dec 30;14(12):e0226959. doi: 10.1371/journal.pone.0226959 (PMC6936774; doi:10.1371/journal.pone.0226959)
Supplement: S2 Table — (DOCX) [file pone.0226959.s010.docx]

Supporting Information

**Bioassay- and Metabolomics-guided Screening of Bioactive Soil Actinomycetes from the Ancient City of Ihnasia, Egypt**

**Mohamed Sebak ^1,2,*^, Amal E. Saafan^2^,** **Sameh AbdelGhani^2^, Walid Bakeer^2^, Ahmed O. El-Gendy^2^, Laia Castaño Espriu^1^, Katherine Duncan^1^,** **RuAngelie Edrada-Ebel^1*^**

^1^ Strathclyde Institute of Pharmacy and Biomedical Sciences, Faculty of Science, University of Strathclyde, Glasgow, UK.

^2^ Microbiology and Immunology Department, Faculty of Pharmacy, Beni-Suef University, Beni-Suef, Egypt.

***Correspondence:**

Mohamed Sebak

E-mail: [Mohamed.sebak@pharm.bsu.edu.eg](mailto:Mohamed.sebak@pharm.bsu.edu.eg)

RuAngelie Edrada-Ebel

E-mail: [Ruangelie.edrada-ebel@strath.ac.uk](mailto:Ruangelie.edrada-ebel@strath.ac.uk)

**S2 Table. Dereplication of selected major ion peaks in MS.REE. 13.**

| Peak no. | MZmine ID | RT (min) | m/z [Ionization] | MW | Predicted Molecular formula | Putative compound identified  /Known source | Peak Area |
| --- | --- | --- | --- | --- | --- | --- | --- |
| 1 | P_11803 | 10.66 | 379.165 [M+H]^+^ | 378.158 | C_22_H_22_N_2_O_4_ | 2'-deoxy, 2'-(dibenzylamino)clavulanic acid; | 3.14E+08 |
|  |  |  |  |  | C_22_H_22_N_2_O_4_ | nocazine A /  marine-derived *Nocardiopsis dassonvillei* HR10-5 |  |
|  |  |  |  |  | C_22_H_22_N_2_O_4_ | (2-[3-hydroxy-2-methoxy-1- (1*H*-indol-3-yl) propyl]-1*H*-indole-3-acetic acid)  /marine-derived *Rubrobacter radiotolerans* |  |
| 2 | P_15581 | 11.92 | 371.154 [M+H]^+^ | 370.146 | C_19_H_22_N_4_O_2_S | 1''-*N*-propanoyl-TM 64; (ξ)-form,  /*Thermoactinomyces sp.* TA66-2 | 1.71E+08 |
| 3 | P_15328 | 14.66 | 330.181 [M+H]^+^ | 329.174 | C_18_H_23_N_3_O_3_ | no hits | 3.07E+08 |
| 4 | P_15267 | 15.16 | 312.170 [M+H]^+^ | 311.163 | C_18_H_21_N_3_O_2_ | carnequinazoline C  /marine-derived fungus *Aspergillus carneus* KMM 4638 | 2.69E+08 |
|  |  |  |  |  | C_18_H_21_N_3_O_2_ | Talathermophilin E/*Talaromyces thermophilus* strain YM3-4 |  |
| 5 | P_22206 | 16.81 | 490.234 [M+H]^+^ | 489.226 | C_28_H_31_N_3_O_5_ | paraherquamide I  /*Penicillium cluniae* (CECT 2888) | 1.64E+08 |
| 6 | N_955 | 17.35 | 488.306  [M-H]^-^ | 489.313 | C_18_H_39_N_11_O_5_  C_17_H_43_N_7_O_9_ | no hits | 1.76E+08 |
