# Supplementary material for: Bioassay- and metabolomics-guided screening of bioactive soil actinomycetes from the ancient city of Ihnasia, Egypt
Source: PLoS One. 2019 Dec 30;14(12):e0226959. doi: 10.1371/journal.pone.0226959 (PMC6936774; doi:10.1371/journal.pone.0226959)
Supplement: S3 Table — (DOCX) [file pone.0226959.s011.docx]

Supporting Information

**Bioassay- and Metabolomics-guided Screening of Bioactive Soil Actinomycetes from the Ancient City of Ihnasia, Egypt**

**Mohamed Sebak ^1,2,*^, Amal E. Saafan^2^,** **Sameh AbdelGhani^2^, Walid Bakeer^2^, Ahmed O. El-Gendy^2^, Laia Castaño Espriu^1^, Katherine Duncan^1^,** **RuAngelie Edrada-Ebel^1*^**

^1^ Strathclyde Institute of Pharmacy and Biomedical Sciences, Faculty of Science, University of Strathclyde, Glasgow, UK.

^2^ Microbiology and Immunology Department, Faculty of Pharmacy, Beni-Suef University, Beni-Suef, Egypt.

***Correspondence:**

Mohamed Sebak

E-mail: [Mohamed.sebak@pharm.bsu.edu.eg](mailto:Mohamed.sebak@pharm.bsu.edu.eg)

RuAngelie Edrada-Ebel

E-mail: [Ruangelie.edrada-ebel@strath.ac.uk](mailto:Ruangelie.edrada-ebel@strath.ac.uk)

**S3 Table. Dereplicated selected major ion peaks in MS.REE. 22.**

| Peak no. | MZmine ID | RT (min) | m/z [Ionization] | MW | Predicted Molecular formula |  | Putative compound identified in DNP/ Known Source | Peak Area |
| --- | --- | --- | --- | --- | --- | --- | --- | --- |
| 1 | P_11499 | 5.94 | 441.188 [M+H]^+^ | 440.181 | C_21_H_24_N_6_O_5_  C_20_H_28_N_2_O_9_ |  | no hits | 8.23E+07 |
| 2 | P_11932 | 6.66 | 204.077 [M+H]^+^ | 203.069 | C_10_H_9_N_3_O_2_ |  | no hits | 8.59E+07 |
| 3 | P_11857 | 8.48 | 328.127 [M+H]^+^ | 327.119 | C_13_H_13_N_9_O_2_  C_12_H_17_N_5_O_6_ |  | no hits | 8.39E+07 |
| 4 | P_11803 | 10.66 | 379.165 [M+H]^+^ | 378.158 | C_22_H_22_N_2_O_4_ |  | 2'-deoxy, 2'-(dibenzylamino)clavulanic acid; | 2.13E+08 |
|  |  |  |  |  | C_22_H_22_N_2_O_4_ |  | nocazine A /  marine-derived *Nocardiopsis dassonvillei* HR10-5 |  |
|  |  |  |  |  | C_22_H_22_N_2_O_4_ |  | (2-[3-hydroxy-2-methoxy-1- (1*H*-indol-3-yl) propyl]-1*H*-indole-3-acetic acid)  /marine-derived *Rubrobacter radiotolerans* |  |
| 5 | N_1897 | 8.12 | 344.118  [M-H]^-^ | 345.125 | C_8_H_15_N_11_O_5_  C_7_H_19_N_7_O_9_ |  | no hits | 6.74E+07 |
| 6 | N_2029 | 16.41 | 431.209  [M-H]^-^ | 432.216 | C_25_H_28_N_4_O_3_  C_24_H_32_O_7_ |  | no hits | 3.67E+07 |
